# P21‐Activated Kinase 2 as a Novel Target for Ventricular Tachyarrhythmias Associated with Cardiac Adrenergic Stress and Hypertrophy

**DOI:** 10.1002/advs.202411987

**Published:** 2025-03-11

**Authors:** Tao Li, Ting Liu, Yan Wang, Yangpeng Li, Leiying Liu, James Bae, Yu He, Xian Luo, Zhu Liu, Tangting Chen, Xianhong Ou, Dan Zhang, Huan Lan, Juyi Wan, Yan Wei, Fang Zhao, Xin Wang, Tao Li, Christopher L.‐H. Huang, Chunxiang Zhang, Ming Lei, Xiaoqiu Tan

**Affiliations:** ^1^ Key Laboratory of Medical Electrophysiology of the Ministry of Education Medical Electrophysiological Key Laboratory of Sichuan Province Institute of Cardiovascular Research Southwest Medical University Luzhou Sichuan 646000 China; ^2^ Department of Cardiology The Affiliated Hospital Southwest Medical University Luzhou Sichuan 646000 China; ^3^ Department of Pharmacology University of Oxford Mansfield Road Oxford OX1 3QT UK; ^4^ Department of Cardiology Zhongnan Hospital of Wuhan University East Lake Road 169 Wuhan 430071 China; ^5^ Faculty of Biology, Medicine and Health The University of Manchester Dover Street Manchester M13 9GB UK; ^6^ Laboratory of Mitochondria and Metabolism West China Hospital of Sichuan University Chengdu 610041 China; ^7^ Physiological Laboratory University of Cambridge Downing Street Cambridge CB2 3EG UK; ^8^ Department of Biochemistry University of Cambridge Tennis Court Road Cambridge CB2 1QW UK; ^9^ Department of Physiology School of Basic Medical Sciences Southwest Medical University Luzhou Sichuan 646000 China

**Keywords:** cardiac arrhythmia, Ca^2+^ handling, mitochondrial oxidative stress, NADPH oxidase, P21‐activated kinase 2

## Abstract

Ventricular arrhythmias associated with cardiac adrenergic stress and hypertrophy pose a significant clinical challenge. We explored ventricular anti‐arrhythmic effects of P21‐activated kinase 2 (Pak2), comparing in vivo and ex vivo cardiomyocyte‐specific Pak2 knockout (Pak2^cko^) or overexpression (Pak2^ctg^) murine models, under conditions of acute adrenergic stress, and hypertrophy following chronic transverse aortic constriction (TAC). Pak2 was downregulated 5 weeks following the latter TAC challenge. Cellular physiological, optical action potential and Ca^2+^ transient, measurements, demonstrated increased incidences of triggered ventricular arrhythmias, and prolonged action potential durations (APD) and altered Ca^2+^ transients with increases in their beat‐to beat variations, in Pak2^cko^ hearts. Electron microscopic, proteomic, and molecular biological methods revealed a mitochondrial localization of stress‐related proteins on proteomic and phosphoproteomic analyses, particularly in TAC stressed Pak2^cko^ mice. They further yielded accompanying evidence for mitochondrial oxidative stress, increased reactive oxygen species (ROS) biosynthesis, reduced mitochondrial complexes I‐V, diminished ATP synthesis and elevated NADPH oxidase 4 (NOX4) levels. Pak2 overexpression and the novel Pak2 activator JB2019A ameliorated these effects, enhanced cardiac function and decreased the frequencies of triggered ventricular arrhythmias. Pak2 activation thus protects against ventricular arrhythmia associated with cardiac stress and hypertrophy, through unique mechanisms offering potential novel therapeutic anti‐arrhythmic targets.

## Graphical abstract

Ca^2+^/calmodulin‐dependent kinase II (CaMKII) activation is likely driven by oxidative stress, particularly excessive reactive oxygen species (ROS) production. This study identifies P21‐activated kinase 2 (Pak2) as a novel regulator of ROS‐induced CaMKII activation and abnormal Ca^2^⁺ dynamics in acute adrenergic and chronic pressure‐overload stressed hearts. Thus, Pak2's regulation of ROS production and Ca^2^⁺ homeostasis may represent a novel therapeutic target for alleviating cardiac ventricular arrhythmias associated with adrenergic stress and pathological hypertrophy.

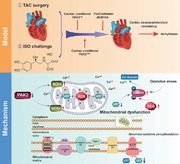

## 1 Introduction

Sudden cardiac death (SCD) accounts for approximately half of all deaths related to cardiovascular disease, posing a significant international public health challenge.^[^ ^1^ ^]^ Malignant ventricular arrhythmias constitute the commonest cause of SCD. These often result from structural and electrical myocardial remodeling in response to structural or functional stress.^[^ ^2^ ^]^ Despite extensive research and development, current interventional anti‐arrhythmic approaches, including use of implantable cardioverter‐defibrillator and ventricular ablation devices, remain limited by their ineffectiveness in addressing the underlying disease pathophysiology.^[^ ^3^, ^4^ ^]^

Previous studies in both human and animal models had implicated mitochondrial dysfunction in arrhythmogenesis.^[^ ^5^, ^6^ ^]^ Associated reductions in ATP synthesis and increased reactive oxygen species (ROS) production cause cellular and ionic malfunction leading to altered automaticity, re‐entry and conduction block.^[^ ^6^, ^7^ ^]^ Increased ROS may also induce cardiac arrhythmias through directly oxidizing and thereby activating Ca^2+^/calmodulin‐dependent kinase II (CaMKII).^[^ ^8^ ^]^ We have previously reported that activation of signaling by the serine‐threonine kinase p21‐activated kinase 1 (Pak1) protects cardiomyocytes against ischemic cell injury and hypertrophic stress.^[^ ^9^, ^10^, ^11^, ^12^, ^13^, ^14^ ^]^ The accompanying sarcolemmal and mitochondrial dysregulation may offer promising therapeutic targets for ameliorating arrhythmia.^[^ ^6^, ^7^ ^]^

Recent new studies have also implicated Pak2 in the cellular regulation of cardiac homeostasis. It confers cardio‐protection and is essential for normal ion channel activity, intracellular Ca^2+^ handling, and cardiac contractility.^[^ ^15^ ^]^ Pak2 is mostly localized near endoplasmic reticulum (ER) and mitochondria. It may regulate the cardioprotective IRE (inositol‐requiring enzyme)1/XBP (X‐box–binding protein)1‐dependent unfolded protein response (UPR).^[^ ^15^, ^16^ ^]^ Mice with cardiac‐specific *Pak2* deletion showed defective ER stress responses, cardiac dysfunction, and profound cardiomyocyte death under conditions of tunicamycin‐induced ER stress or pressure overload. Their gene array analysis then implicated the IRE1/XBP1‐dependent pathway in this Pak2‐mediated regulation of protective and appropriate ER stress responses. Contrastingly, inducing Pak2 activation through genetic overexpression or viral gene delivery improved ER function and cardiac performance. It reduced apoptosis and protected from heart failure.^[^ ^15^, ^16^ ^]^ These findings implicate Pak2‐mediated modulation of ER stress as cardioprotective in cardiac hypertrophy.

Currently, there is no direct evidence bearing on possible protective roles of Pak2 in mitochondrial oxidative stress and cardiac injury. The present study explored possible cardioprotective effects of Pak2 on the incidence and generation of ventricular arrhythmias. Cardiomyocyte‐specific Pak2 knockout (*Pak2* ^cko^) or Pak2 overexpression (*Pak2* ^ctg^) mice were studied under acute adrenergic, isoproterenol‐induced, and chronic transverse aortic constriction (TAC) induced pressure overload. Related electrophysiological, Ca^2+^ homeostatic, cardiac hypertrophic, metabolic and energetic changes were demonstrated at both the whole organ and cellular levels. Furthermore, a Pak2 activator JB2019A was also assessed for its anti‐hypertrophic and anti‐ventricular arrhythmic effects.

Together the findings suggest that modulating mitochondrial function and ROS production through targeting Pak2 may offer a strategy for managing ventricular arrhythmias and preventing SCD. We corroborate these findings developing and utilizing a direct Pak2 activator and demonstrating its cardioprotective effects. Pak2 thus emerges as a promising therapeutic kinase target for the development of novel effective anti‐arrhythmic drugs.

## 2 Results

### 2.1 Pak2 Deficiency Disrupts Ca^2+^ Homeostasis and Increases Susceptibility to Ventricular Arrhythmias

**Figure 1:**
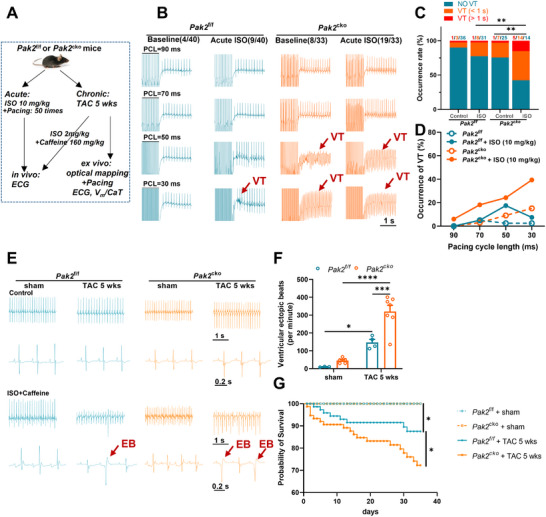
Pak2 deficiency aggravates the inducibility of cardiac arrhythmias in mice challenged with acute isoproterenol (ISO) or chronic (5 weeks) TAC challenge in vivo. A) The protocol scheme applied to Pak2^f/f^ and Pak2^cko^ mice under acute isoproterenol (ISO) or chronic TAC challenge. B) Representative in vivo cardiac electrophysiological recordings from Pak2^f/f^ and Pak2^cko^ mice with different frequencies of burst stimulation. C) VT inducibility during in vivo electrocardiogram measurements in Pak2^f/f^ and Pak2^cko^ mice before and after isoproterenol challenge. D) Quantification of VT occurrence following the application of isoproterenol in Pak2^f/f^ and Pak2^cko^ mice at different frequencies of burst stimulation. E) Representative in vivo cardiac electrophysiological recordings from Pak2^f/f^ and Pak2^cko^ mice at 5 weeks after sham or TAC surgery. F) Occurrence of ventricular ectopic beats of Pak2^f/f^ and Pak2^cko^ mice at 5 weeks after sham or TAC surgery. G) Survival curves of four groups of mice after operation. *p < 0.05, **p < 0.01, ***p < 0.001, ****p < 0.0001.

We first investigated potential protective effects of Pak2 signaling on ventricular arrhythmogenic tendency under conditions of acute isoproterenol induced cardiac adrenergic stress and chronic TAC induced ventricular hypertrophy. We compared electrical stability and Ca^2+^ handling in hearts of Pak2 deficient (*Pak2* ^cko^) and flox (*Pak2* ^f/f^) mice, in an absence (*Pak2* ^cko^ and *Pak2* ^f/f^ groups) and 5‐weeks following chronic hypertrophic stress brought about by transverse aortic constriction (TAC) (*Pak2* ^cko^/TAC and *Pak2* ^f/f^/TAC groups) both in in vivo animals and *ex vivo* whole hearts (**Figure** **1**A). Intact *Pak2* ^cko^ (n = 11) and *Pak2* ^f/f^ (n = 13) mice exhibited similar baseline heart rates (HR), electrocardiographic P wave durations, P‐R intervals, QRS durations, but there were prolonged QT intervals in *Pak2* ^cko^ mice (Figure 1A and Table S3, Supporting Information). Following (5 min) isoproterenol induced acute adrenergic stress, *Pak2* ^cko^ mice showed increased incidences, 57.58% (19/33), of ventricular tachyarrhythmias (VT) relative to 22.5% (9/40) of untreated controls, particularly at higher pacing frequencies (Figure 1B–D). 5‐weeks following TAC, expression of Pak2 was significantly downregulated (Figure S2, Supporting Information). *Pak2* ^cko^/TAC mice then showed an exacerbated cardiac hypertrophy and dysfunction (Figure S2, Supporting Information). They showed increased occurrences of ventricular ectopic beats and VT/VF (n = 7) compared to *Pak2* ^f/f^/TAC mice (n = 4) on challenge with isoproterenol and caffeine (Figure 1E,F). Survival curve analyses associated the combination of Pak2 deficiency and TAC challenge with significantly reduced overall survival rates (*P* < 0.05, Figure 1G).

**Figure 2:**
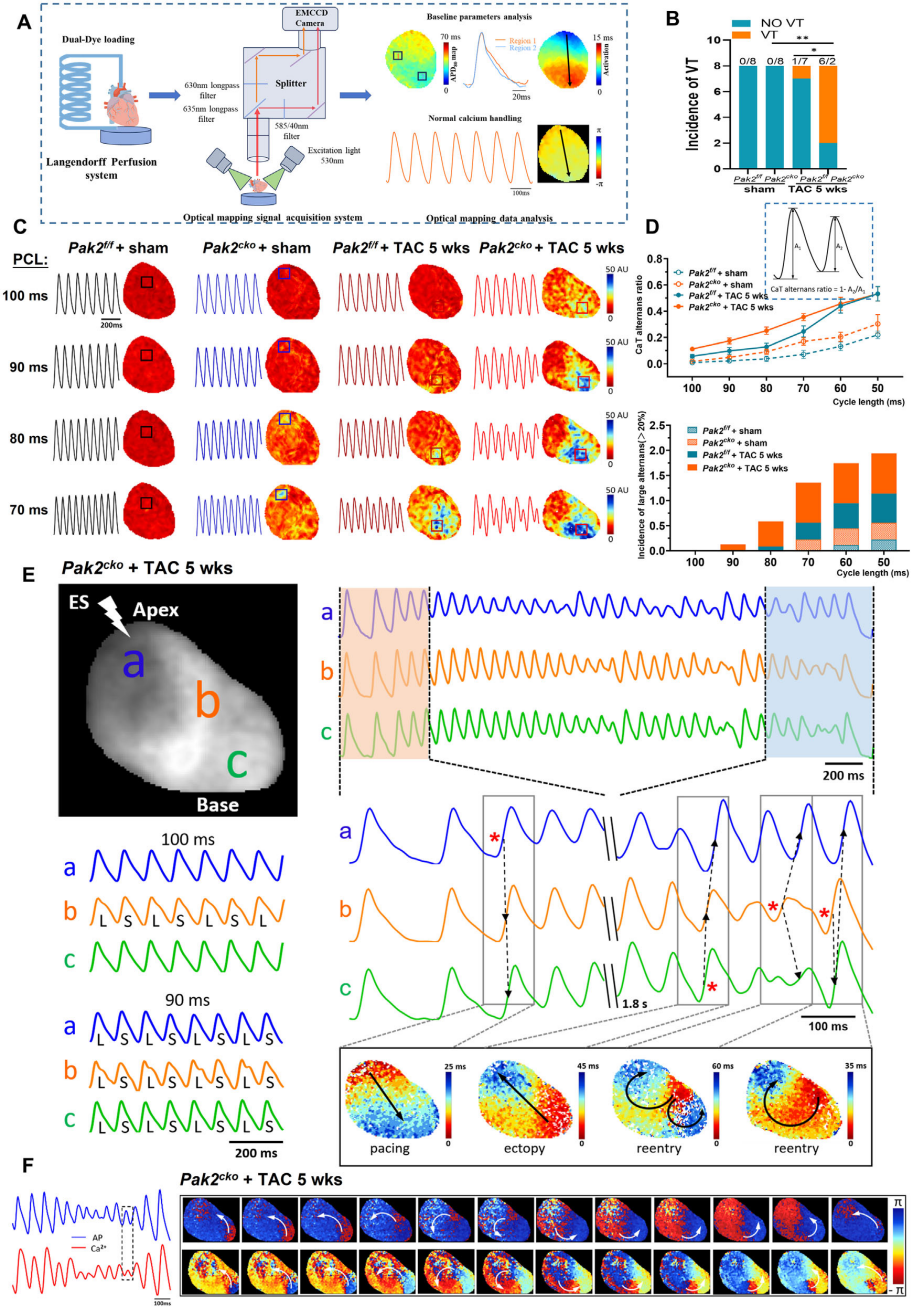
Pak2 deficiency aggravates the inducibility of VT and alternans in mice under chronic (5 weeks) TAC challenge in ex vivo hearts. A) Flow chart of optical mapping scheme. B) VT inducibility in ex vivo Langendorff hearts after TAC challenge in Pak2^f/f^ and Pak2^cko^ mice. C) Heat maps (indicating the spectral intensity differences of Ca^2+^ transients) showing frequency‐dependent CaT alternans during progressive decrements in cycle length between 100–70 ms. The corresponding CaT traces are also exhibited from the selected region within the boxes. D) Calculation of CaT alternans ratio for each group (n = 6 hearts for each group). Large alternans ratio (>20%) are counted for evaluation of the severity of CaT alternans. E) Typical AP traces during VT induced by progressive pacing; the activation map shows that the initiation positions for ectopy and reentry overlap with regions harboring the most severe AP alternans. F) Typical consecutive phase maps during VT in a Pak2^cko^ mouse heart, showing the fast re‐entry rotors meandering on the epicardium without losing the coupling of AP and CaT, along with the corresponding AP and CaT traces. *p < 0.05, **p < 0.01, ***p < 0.001.

Further investigations compared isolated Langendorff perfused *Pak2* ^cko^ (n = 8) and *Pak2* ^f/f^ (n = 8) hearts using combined high‐resolution dual‐dye optical mapping of membrane potentials (*V_m_*) and Ca^2+^ transients (CaT) (**Figure** **2**). Both *Pak2* ^cko^ and *Pak2* ^f/f^ hearts showed no significant baseline differences in their incidences of ventricular arrhythmias (Figure 2B). However, acute isoproterenol challenge selectively increased VT incidences in the *Pak2* ^cko^ hearts (Figure S3D, Supporting Information). Similarly, only 1 out of 8 *Pak2* ^f/f^/TAC hearts, but 6 out of 8 *Pak2* ^cko^/TAC hearts showed ventricular arrhythmias (*P* < 0.05, n = 8 hearts for each group) (Figure 2B). *Pak2* ^cko^ hearts showed increased durations of both action potentials (APD) and Ca^2+^ transients, quantified as APD_80_ and CaTD_50_. These effects were accentuated in *Pak2* ^cko^/TAC hearts (Figure S3A,B, Supporting Information). These findings parallel prolongations in the corresponding in vivo QT intervals (Table S3, Supporting Information). These findings were corroborated by patch clamp electrode action potential (AP) recordings from isolated single cardiomyocytes from the different groups. In full and detailed agreement with the optical mapping results, APD_80_ was increased in *Pak2* ^cko^ relative to *Pak2* ^f/f^, *Pak2* ^cko^/TAC to *Pak2* ^f/f^/TAC as well as *Pak2* ^cko^ to *Pak2* ^cko^/TAC and *Pak2* ^f/f^ to *Pak2* ^f/f^/TAC cardiomyocytes (Figure S3C, Supporting Information).

Further investigations of TAC‐induced arrhythmic tendency explored occurrences and characteristics of CaT alternans (Figure 2C,D; Figure S3E, Supporting Information), *V*_m_/CaT latencies (Figure S4, Supporting Information) and for re‐entry rotors (Figure 2E,F). Typical CaT alternans are illustrated for each group using pacing cycle lengths (PCL) between 100 ms and 70 ms (Figure 2C). CaT alternans showed significantly higher occurrences in *Pak2* ^cko^/TAC (n = 6) compared to *Pak2* ^f/f^/TAC hearts (n = 6). CaT alternans analysis indicated that rapid pacing, between PCLs of 100 and 50 ms, increased CaT alternans ratios and incidences of amplitude alternans (>20%). Pak2 deficiency significantly aggravated the frequencies of this alternans (Figure 2D). Both TAC (Figure S3E, Supporting Information), and Pak2 deficiency also accentuated the effects of acute isoproterenol challenge in increasing frequencies of CaT alternans. Finally, examining *V*_m_/CaT latencies demonstrating the coupling of *V*_m_ and [Ca^2+^]_i_ dynamics showed that Pak2 deficiency aggravated TAC‐induced increments of *V*_m_/CaT latency (Figure S4A,B, Supporting Information).

*Pak2* ^cko^/TAC hearts also exhibited APD alternans, and this led to ectopic beats and rotor and re‐entry formation (Figure 2E,F). The membrane potential (*V_m_*) traces demonstrated APD alternans typically occurring at PCLs ≈90 ms. Further analysis of AP conduction and the formation of VT revealed that APs normally conducted in a direction from apex to base (a→b→c). In contrast, when an ectopic beat occurred at site c, the conduction route reversed in direction running from c to a (c→b→a). When the ectopic beat occurred at b, the conduction direction altered to either b to a or b to c (b→a/c or b→c→a), creating a re‐entry arrhythmic route (Figure 2E).

### 2.2 Pak2 Overexpression Rescues Isoproterenol or TAC‐Induced Cardiac Arrhythmias

**Figure 3:**
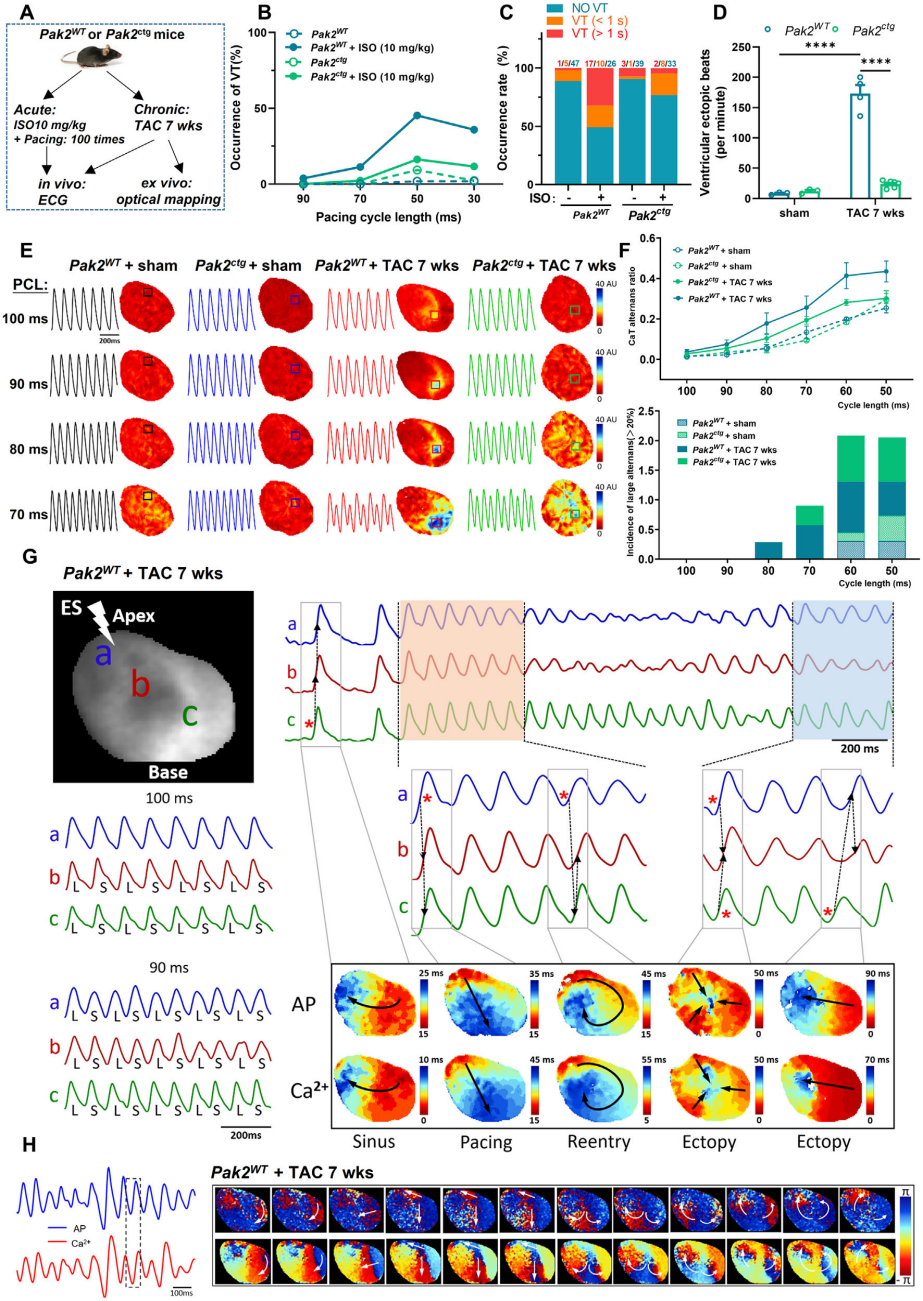
Cardiac specific Pak2 overexpression attenuates isoproterenol or TAC‐induced cardiac arrhythmias and Ca^2+^ alternans. A) The protocol scheme for Pak2^WT^ (Rosa26^CAG‐LSL‐Pak2^) and Pak2^ctg^ mice with acute isoproterenol (ISO) or chronic TAC challenge. B) VT inducibility during in vivo electrocardiogram measurement in Pak2^WT^ and Pak2^ctg^ mice before and after isoproterenol challenge with rapid pacing for 100 stimuli each. C) Quantification of VT duration in the application of isoproterenol in Pak2^WT^ and Pak2^ctg^ mice in vivo. D) Calculation of ventricular ectopic beats after TAC challenge for 5 weeks in WT and Pak2^ctg^ mice in vivo with isoproterenol and caffeine challenge. E) Heat maps (indicating the spectral intensity difference of Ca^2+^ alternans) showing frequency‐dependent CaT alternans during progressive decrements of pacing cycle length between 100–70 ms. Corresponding CaT traces are also exhibited from the selected region within the boxes. F) Calculation of CaT alternans ratio for each group (n = 4‐7 hearts). Large alternans ratio (>20%) are counted for evaluation of the severity of CaT alternans. G) Typical AP traces during VT in a Pak2^WT^ mouse heart after TAC for 7 weeks induced by a progressive pacing; activation map shows the initiation positions of ectopy and reentry overlap with a region harboring the most severe AP alternans. H) Typical consecutive phase maps during VT, showing the fast reentry rotors meandering on the epicardium without losing the coupling of AP and CaT, along with the corresponding AP and CaT traces. *p < 0.05, **p < 0.01, ***p < 0.001, ****p < 0.0001.

We then developed a mouse *Pak2* ^ctg^ model with inducible cardiac‐specific overexpression of wild type Pak2 driven by Myh6‐creERT (Figure S1, Supporting Information) to explore effects of Pak2 overexpression on acute isoproterenol or TAC‐induced ventricular arrhythmias (**Figure** **3**; Figure S5, Supporting Information). Baseline echocardiographic analyses suggested normal cardiac function in both *Pak2* ^ctg^ and control *Pak2* ^WT^ (Rosa26^CAG‐LSL‐Pak2^) mice (Figure S5C,D, Supporting Information). Cardiac *Pak2* ^ctg^ mice exhibited reduced incidences and durations of isoproterenol‐induced ventricular arrhythmias (Figure 3B,C; Figure S5E, Supporting Information). TAC, here applied for 7 weeks, increased the frequency of ventricular ectopic beats from 8.33 ± 1.20 to 173.00 ± 14.11 (*P* < 0.001) in control *Pak2* ^WT^ mice, while Pak2 overexpression alleviated the effect of TAC with decreasing ventricular ectopic beats to 39.23 ± 2.07 (*P* < 0.001) in the *Pak2* ^ctg^/TAC groups (Figure 3D; Figure S5F, Supporting Information). Pak2 activation also suppressed pressure overload–induced hypertrophy. Thus, wild type (WT) WT/TAC but not *Pak2* ^ctg^/TAC mice showed increases in left ventricular mass and hypertrophy (Figure S5A,B, Supporting Information) and left ventricular end‐diastolic diameters, left ventricular end‐systolic diameter, and decreased fraction shortening on echocardiographic analyses (Figure S5C,D, Supporting Information) relative to unoperated controls.

Finally *Pak2* ^ctg^/TAC hearts did not show remodeled *V_m_* and Ca^2+^ optical mapping (Figure 3E–H; Figure S5, Supporting Information), Ca^2+^ transient alternans, including CaT alternans ratios and incidences (>20%) (Figure 3E,F) shown by *Pak2* ^cko^ hearts (Figure 2C,D). In contrast, progressively paced WT/TAC hearts showed AP traces suggesting rotor formation and re‐entry phenomena (Figure 3G) and *V_m_*/CaT heat maps (Figure 3H) suggesting pro arrhythmic abnormal Ca^2+^ handling and coupling with *V_m_*.

### 2.3 Isolated Single Pak2^cko^ Ventricular Myocytes Show Abnormal Ca^2+^ Dynamics

**Figure 4:**
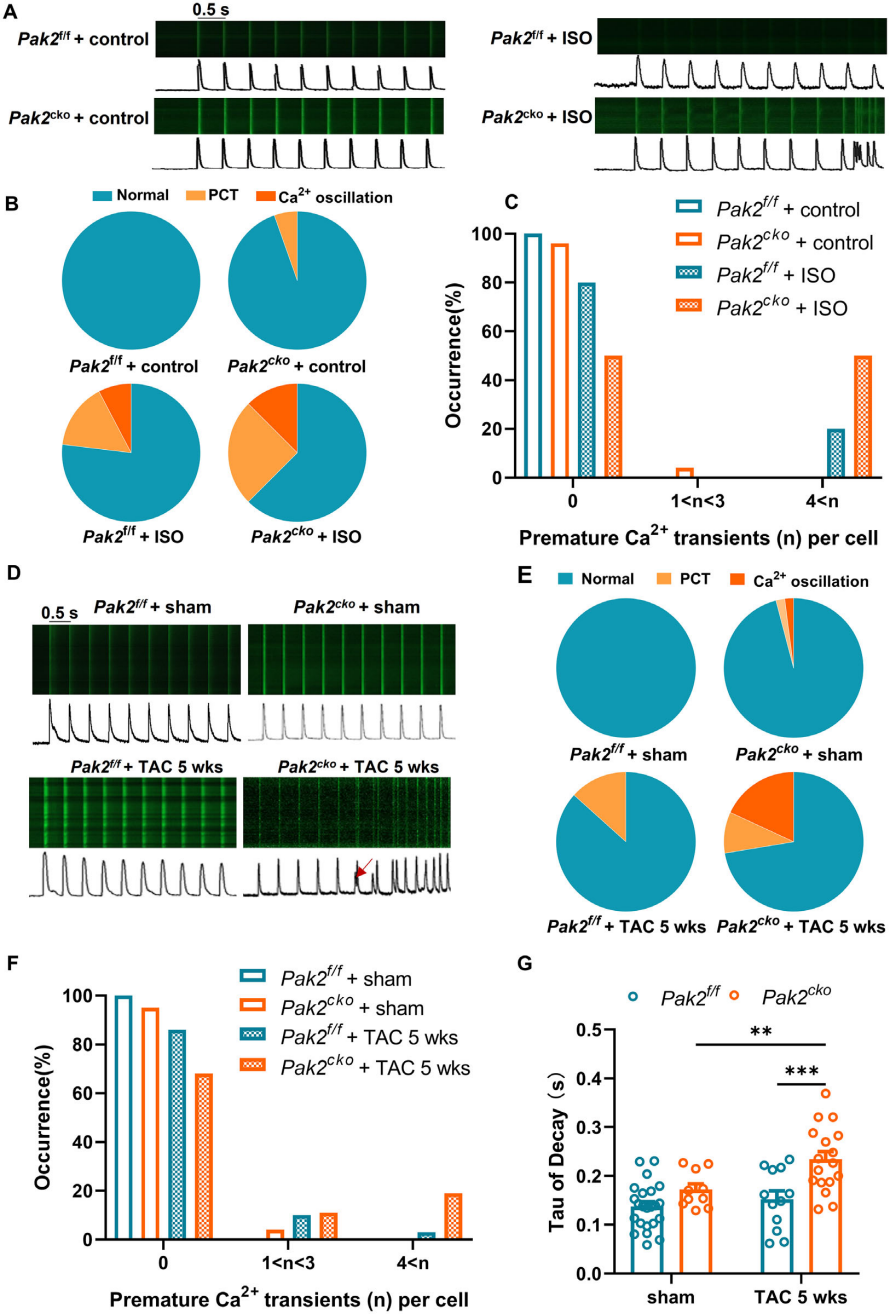
Pak2 deletion aggravates isoproterenol and TAC‐induced disruptions of cardiac intracellular calcium homeostasis. A) Calcium fluorescence was measured in isolated ventricular myocytes from Pak2^f/f^ and Pak2^cko^ mice after acute isoproterenol treatment. B) The pie diagrams of occurrence of premature Ca^2+^ transients (PCT) and Ca^2+^ oscillation (CO) from the indicated groups. C) The statistical histogram of severity of Ca^2+^ transient abnormality from the indicated groups. D) Calcium fluorescence was measured in isolated cardiomyocytes from Pak2^f/f^ and Pak2^cko^ mice after sham or TAC surgery for 5 weeks. E) The pie diagrams of occurrence of PCT and CO from the indicated groups. F) The statistical histogram of severity of Ca^2+^ transient abnormality from the indicated groups. G) The statistical histogram of τ of Ca^2+^ transient decays from the indicated groups. *p < 0.05, **p < 0.01, ***p < 0.001.

We next studied Ca^2+^ transients in *Pak2* ^cko^, *Pak2* ^cko^/TAC, *Pak2* ^f/f^ and *Pak2* ^f/f^/TAC ventricular myocytes (**Figure** **4**). The *Pak2* ^cko^ myocytes from both acute isoproterenol and chronic 5‐week TAC challenged hearts showed higher incidences of spontaneous Ca^2+^ transients (SCT) and Ca^2+^ oscillations (CO) than the corresponding controls (Figure 4A–C). Additionally, myocytes from *Pak2* ^cko^/TAC hearts showed significantly prolonged Ca^2+^ transient decay time constants (Figure 4D–G).

### 2.4 Defective Mitochondrial Biosynthesis and Oxidative Phosphorylation in Pak2^cko^ Hearts

The above results implicated Pak2 in maintaining cardiac electrical stability and Ca^2+^ homeostasis. To determine the underlying signaling pathways, we conducted proteomics and phosphoproteomics analyses in ventricular tissues from *Pak2* ^cko^, *Pak2* ^cko^/TAC, *Pak2* ^f/f^ and *Pak2* ^f/f^/TAC mice.

**Figure 5:**
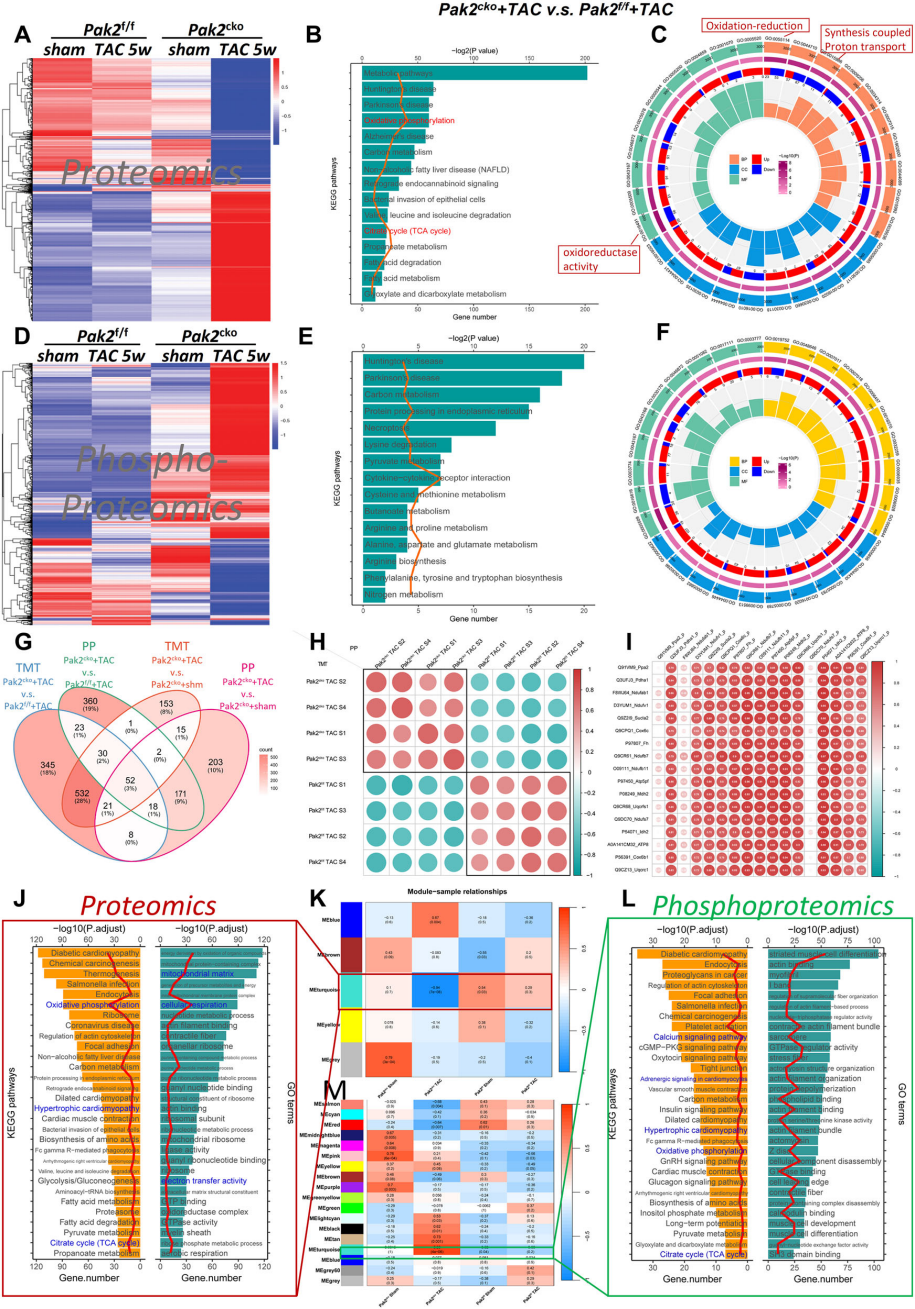
Pak2 deletion aggravates TAC‐induced cardiac oxidative stress. A) Heat map showing differentially expressed protein with proteomics in heart tissue from Pak2^f/f^ and Pak2^cko^ mice with sham or TAC surgery for 5 weeks. B) KEGG enrichment analysis of differentially expressed protein from Pak2^f/f^ and Pak2^cko^ mice at 5 weeks after TAC surgery. C) GO enrichment analysis of proteomics in heart tissue from different treatments. D) Heat map showing differentially expressed protein with phos‐proteome in heart tissue from different treatments. E) KEGG enrichment analysis of phos‐proteomics in heart tissue from different treatments. F) GO enrichment analysis of phos‐proteomics. G) Venn graph showing the significantly different proteins and phosphoprotein for Pak2^cko^/TAC versus Pak2^f/f^/TAC, and Pak2^cko^/TAC versus Pak2^cko^/sham respectively. H) Comparing the correlation between the proteomic and phosphoproteomic changes for TMT and PP proteomics results of each sample between Pak2^cko^/TAC and Pak2^f/f^/TAC. I) the relevant proteins enriched in the oxidative phosphorylation and tricarboxylic acid cycle pathways were analyzed for correlation between the phosphoproteomics and proteomics. K,M) The WGCNA method used to identify protein modules associated with sample phenotypes for proteomic (K) and phosphoproteomic arrays (M). J,L) The turquoise module, which is most associated with the sample phenotype, was subjected to KEGG and GO pathway enrichment analysis for proteomic (J) and phosphoproteomic arrays (L).

First, multiple group comparison of the proteomes was performed for the distinct *Pak2* ^f/f^ versus *Pak2* ^cko^ genotypes in sham versus TAC conditions (Table S4, Supporting Information). Of 4203 screened expressed proteins, 1268 differentially expressed proteins were already associated with cardiac stress. *Pak2* ^cko^/TAC mice exhibited elevated levels of such stress associated proteins, reflected in their highest proteomic and phosphoproteomic changes (**Figure** **5**A,D). KEGG and GO enrichment studies revealed enriched oxidative phosphorylation related metabolic pathways in *Pak2* ^cko^/TAC relative to *Pak2* ^f/f^/TAC hearts in the proteomics (Figure 5B,C) and phosphoproteomics arrays (Figure 5E,F). Further analysis (Figure 5G) showed that each set represents significantly different proteins and phosphoproteins for *Pak2* ^cko^/TAC versus *Pak2* ^f/f^/TAC, and *Pak2* ^cko^/TAC versus *Pak2* ^cko^ respectively. Of these, 123 proteins overlapped between the *Pak2* ^cko^/TAC and *Pak2* ^f/f^/TAC groups and 90 proteins overlapped between the *Pak2* ^cko^/TAC and *Pak2* ^cko^ groups. Comparing the TMT and PP proteomics results suggest a marked correlation between the proteomics and phosphoproteomics changes between *Pak2* ^cko^/TAC and *Pak2* ^f/f^/TAC groups (Figure 5H). After performing enrichment analysis on the significantly different proteins in the proteomics and phosphoproteomics of the *Pak2* ^cko^/TAC versus *Pak2* ^f/f^/TAC groups, the relevant proteins enriched in the oxidative phosphorylation and tricarboxylic acid cycle pathways were analyzed for correlations between the phosphoproteomics and proteomics. The results indicate that the expression correlation of these proteins is also consistent (Figure 5I).

The Weighted Gene Co‐Expression Network Analysis (WGCNA) method was used to identify protein modules associated with sample phenotypes. First, the proteomic data of the four groups were preprocessed, filtering out low‐expression proteins and standardizing the remaining proteins. A soft threshold power β = 6 was selected to construct the weighted network, ensuring the network's scale‐free property. Through hierarchical clustering analysis, five protein modules were identified, each containing highly correlated proteins in the co‐expression analysis. These modules were named with different colors, such as the blue module, yellow module, etc. It was found that the turquoise module was significantly associated with phenotype severity (r = 0.94, P < 0.01) (Figure 5K). The turquoise module, the most associated with the sample phenotype in the *Pak2* ^cko^/TAC group, was then subjected to KEGG and GO pathway enrichment analysis. These pathways indicated associations with cardiomyopathy, oxidative phosphorylation, and other related processes (Figure 5J). The WGCNA method was also used to identify phosphorylated protein modules associated with sample phenotypes across the four groups. Through hierarchical clustering analysis, 18 protein modules were identified. Correlation analysis between these modules and sample phenotype data revealed that the turquoise module was significantly associated with phenotype severity (r = 0.89, *P* < 0.01) (Figure 5M). The turquoise module, most correlated with the sample phenotype in the *Pak2* ^cko^/TAC group, was subjected to KEGG and GO pathway enrichment analysis. The pathways indicated associations with cardiomyopathy and the tricarboxylic acid cycle (Figure 5L).

**Figure 6:**
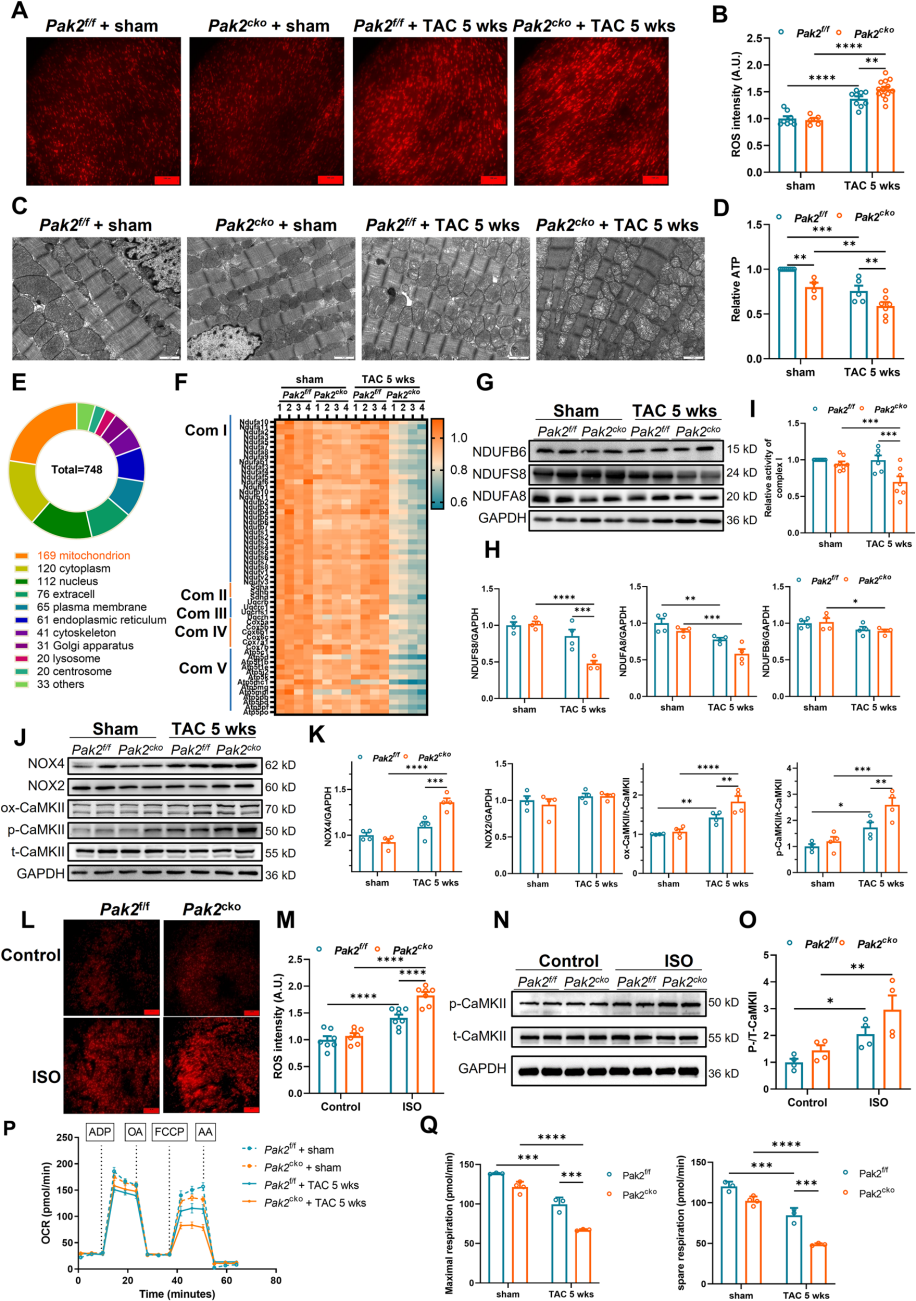
Pak2 deletion aggravates cardiac dysfunction by impairing mitochondrial function. A) Representative fluorescence images shows ROS levels of Pak2^f/f^ and Pak2^cko^ mice at 5 weeks after sham or TAC surgery. Scale bar 100 µm. B) Quantification of panel A. C) Representative transmission electron microscopy images of Pak2^f/f^ and Pak2^cko^ mice at 5 weeks after sham or TAC surgery. Scale bar 1 µm. D) ATP content in Pak2^f/f^ and Pak2^cko^ mice at 5 weeks after sham or TAC surgery. E) Pie diagrams showing the subcellular location of differentially expressed protein in Pak2^f/f^ and Pak2^cko^ mice at 5 weeks after TAC surgery in the comparison between the Pak2^cko^/TAC and Pak2^f/f^/TAC groups. F) Heat map showing differentially expressed mitochondrial respiratory electron transport chain protein in Pak2^f/f^ and Pak2^cko^ mice at 5 weeks after sham or TAC surgery. G) Representative immunoblotting images showing NDUFB6, NDUFS8, NDUFA8 protein from indicated groups. H) Quantification of G (n = 4 mice for each group). I) Statistical graph of relative activity of mitochondrial complex I. J) Representative immunoblotting images showing NOX4, NOX2, ox‐CaMKII, p‐CaMKII and t‐CaMKII protein from indicated groups. K) Quantification of J (n = 4 mice for each group). L) Representative fluorescence images shows ROS levels of Pak2^f/f^ and Pak2^cko^ mice after 30 min isoproterenol treatment. Scale bar 100 µm. M) Quantification of L. N,O) Representative immunoblotting images and statistical graph showing the effect of acute isoproterenol on p‐CaMKII in Pak2^f/f^ and Pak2^cko^ mice (n = 4 mice for each group). P,Q) OCRs were analyzed by seahorse extracellular flux analyses involving sequential injection of ADP, oligomycin (OA), trifluorocarbonyl cyanide phenylhydrazone (FCCP), and antimycin A (AA). *p < 0.05, **p < 0.01, ***p < 0.001, ****p < 0.0001.

We further investigated oxidative stress and mitochondrial structure and function in each group (**Figure** **6**). ROS levels in the *Pak2* ^f/f^/TAC and *Pak2* ^cko^/TAC groups were significantly higher than in the corresponding sham groups (*P* < 0.0001). This associates TAC‐induced chronic cardiac stress with higher ROS damage. Furthermore, the *Pak2* ^cko^/TAC showed higher ROS levels than *Pak2* ^f/f^/TAC hearts (Figure 6A,B). Mitochondrial structure was observed using transmission electron microscopy (TEM) (Figure 6C). Dysmorphic mitochondrial structures were more prominent in *Pak2* ^cko^/TAC cardiomyocytes compared to the corresponding controls. ATP biosynthesis was significantly decreased in the *Pak2* ^cko^/TAC group (Figure 6D), in parallel with the mitochondrial morphological changes.

Differential proteomics analysis demonstrated that the most enriched subcellular location of these stress related proteins was within mitochondria (Figure 6E; Figure S6, Supporting Information). The differential protein numbers in mitochondria were 112 (19.28% of total proteins) in *Pak2* ^cko^/TAC compared to *Pak2* ^cko^ mice, and 169 (22.59% of total differential proteins) in *Pak2* ^cko^/TAC mice compared to *Pak2* ^f/f^/TAC mice (Figure 6E; Figure S6, Supporting Information). Further analysis suggested that mitochondria ETC complex I‐V were significantly decreased in *Pak2* ^cko^/TAC hearts compared to the remaining three groups (Figure 6F). Expression and activity of Complex I (NDUFs) were significantly down‐regulated in *Pak2* ^cko^/TAC hearts (Figure 6G–I). Additionally, the deficiency of Pak2 alone is shown to be insufficient to cause significant damage.

### 2.5 NOX4/ROS Mediated Activation of the CaMKII Pathway Contributes to the Effects of Pak2 on TAC‐Induced Cardiac Electrical Remodeling

The electrophysiological experiments above demonstrated abnormal intracellular Ca^2+^ dynamics and their coupling to *V_m_*. This was further investigated comparing expression of CaMKII and the NOXs, NOX2 and NOX4, related to oxidative stress pathways, between *Pak2* ^cko^, *Pak2* ^f/f^, *Pak2* ^cko^ /TAC and *Pak2* ^f/f^/TAC hearts. NOXs are major main sources of cardiomyocyte ROS production and *Pak2* knockout had previously been reported to influence the NOX4/ROS pathway.^[^ ^17^, ^18^ ^]^ NOX4 was significantly increased in the *Pak2* ^cko^/TAC hearts, in comparison with either *Pak2* ^cko^ or *Pak2* ^f/f^/TAC hearts (Figure 6J,K). NOX2 expression was similar between the different groups. Expression of oxidative and phosphorylated CaMKII (ox‐CaMKII and p‐CaMKII) and correspondingly, ROS levels, were increased in *Pak2* ^cko^/TAC mice compared to *Pak2* ^cko^ and higher in *Pak2* ^f/f^/TAC than *Pak2* ^f/f^ (Figure 6J,K). Similarly, ROS levels in acutely isoproterenol‐challenged *Pak2* ^cko^ hearts were significantly higher than that of acutely isoproterenol‐challenged *Pak2* ^f/f^ and of unchallenged *Pak2* ^cko^ hearts. Thus, Pak2 knockout aggravated the effects of isoproterenol challenge on NOX4, CaMKII and ROS production (Figure 6L–O). Oxygen consumption rate (OCR) was measured as a parameter of mitochondrial respiration activity based on the quantification of oxygen consumption. The activity of mitochondrial respiration was measured by the sequential addition of ADP, oligomycin (OA), Carbonyl cyanide‐4 (trifluoromethoxy) phenylhydrazone (FCCP) and antimycin A (AA). The maximal respiration level and spare respiration of OCR were significantly decreased by TAC treatment relative to sham. Moreover, Pak2 knockout aggravated TAC‐induced mitochondrial dysfunction (Figure 6P,Q).

### 2.6 Pak2 Overexpression Rescues Isoproterenol and TAC‐Induced Mitochondrial Structural and Functional Damage

**Figure 7:**
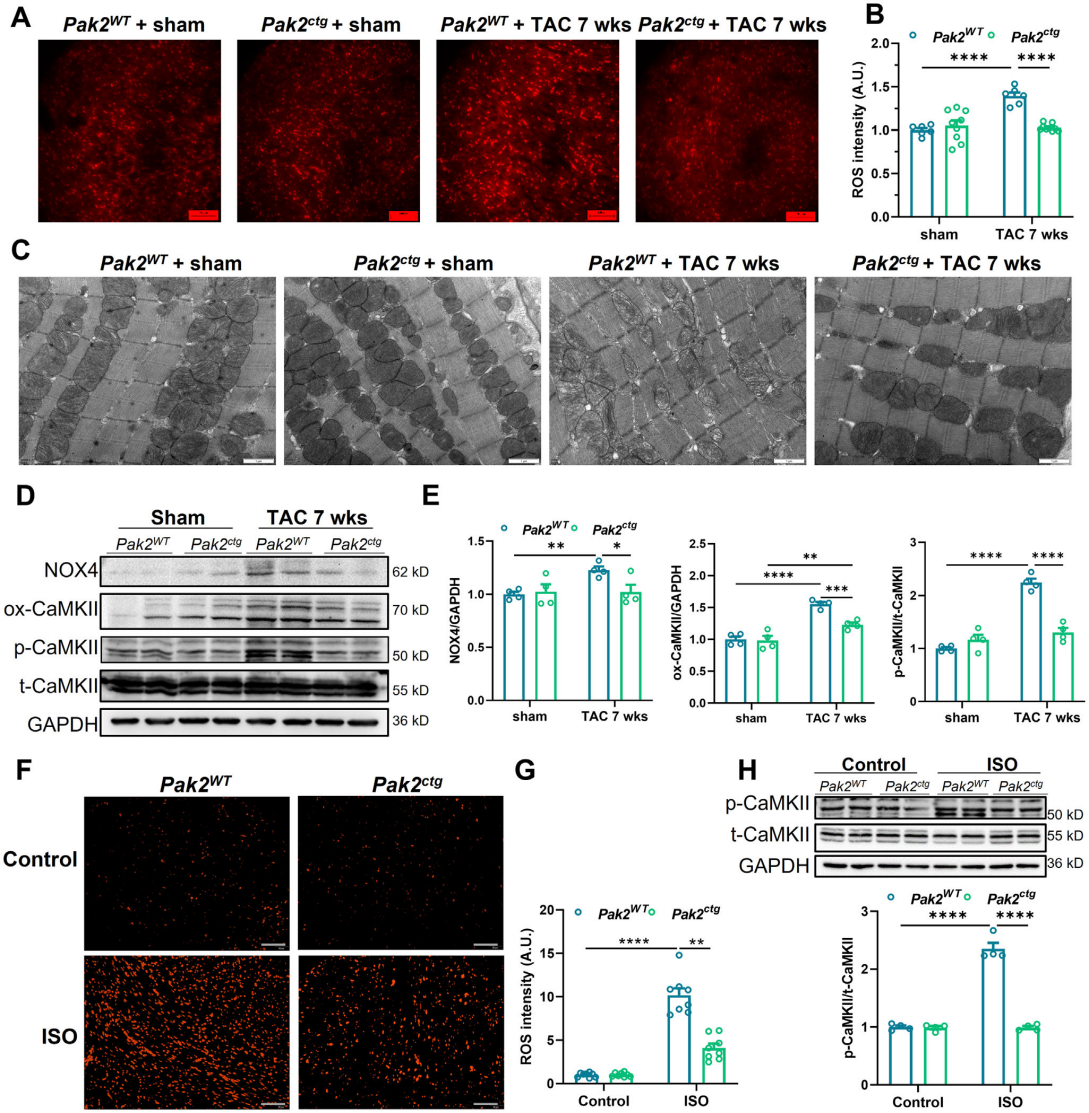
Cardiac‐specific Pak2 overexpression attenuates TAC or isoproterenol‐induced cardiac oxidative stress and abnormalities in mitochondrial structure. A,B) Representative fluorescence images (A) and statistical graph (B) shows ROS levels of Pak2^WT^ and Pak2^ctg^ mice at 7 weeks after sham or TAC surgery. Scale bar 100 µm. C) Representative transmission electron microscopy images of Pak2^WT^ and Pak2^ctg^ mice at 7 weeks after sham or TAC surgery. Scale bar 1 µm. D) Representative immunoblotting images showing NOX4, ox‐CaMKII, p‐CaMKII and t‐CaMKII protein in Pak2^WT^ and Pak2^ctg^ mice at 7 weeks after sham or TAC surgery. E) Quantification of D (n = 4 mice for each group). F,G) Representative fluorescence images (F) and statistical graph (G) shows ROS levels of Pak2^WT^ and Pak2^ctg^ mice at 7 weeks after sham or TAC surgery. Scale bar 100 µm. H) Representative immunoblotting images and statistical analysis showing p‐CaMKII and t‐CaMKII protein in Pak2^WT^ and Pak2^ctg^ mice after sham or acute isoproterenol treatment (n = 4 mice for each group). *p < 0.05, **p < 0.01, ***p < 0.001, ****p < 0.0001.

Experiments comparing *Pak2* ^ctg^, *Pak2* ^ctg^/TAC, *Pak2* ^WT^ and *Pak2* ^WT^/TAC demonstrated that Pak2 overexpression reduced ROS production and ameliorated mitochondrial changes induced by TAC in contrast to findings in *Pak2* ^cko^ (**Figure** **7**A,B). *Pak2* ^ctg^/TAC cardiomyocytes showed reduced numbers of disordered and swollen mitochondria structures compared to *Pak2* ^cko^/TAC cardiomyocytes (Figure 7C). We investigated the possible underlying involvement of the NOX4/ROS/CaMKII pathway in these cardioprotective effects of Pak2 overexpression. *Pak2* ^ctg^/TAC showed lower NOX4, ox‐ and p/t‐CaMKII expression than *Pak2* ^WT^/TAC cardiomyocytes (Figure 7D,E). Finally, acute isoproterenol challenged elicited lower increases in ROS production and p‐CaMKII expression in *Pak2* ^ctg^ than in P*ak2* ^WT^ hearts, even at doubled isoproterenol concentrations (Figure 7F–H).

### 2.7 The Pak2 Activator JB2019A Rescues the Pro‐Arrhythmic Effects of Acute Isoproterenol Challenge and Chronic TAC‐Induced Cardiac Hypertrophy, Reducing Mitochondrial Structural Damage and ROS Production

**Figure 8:**
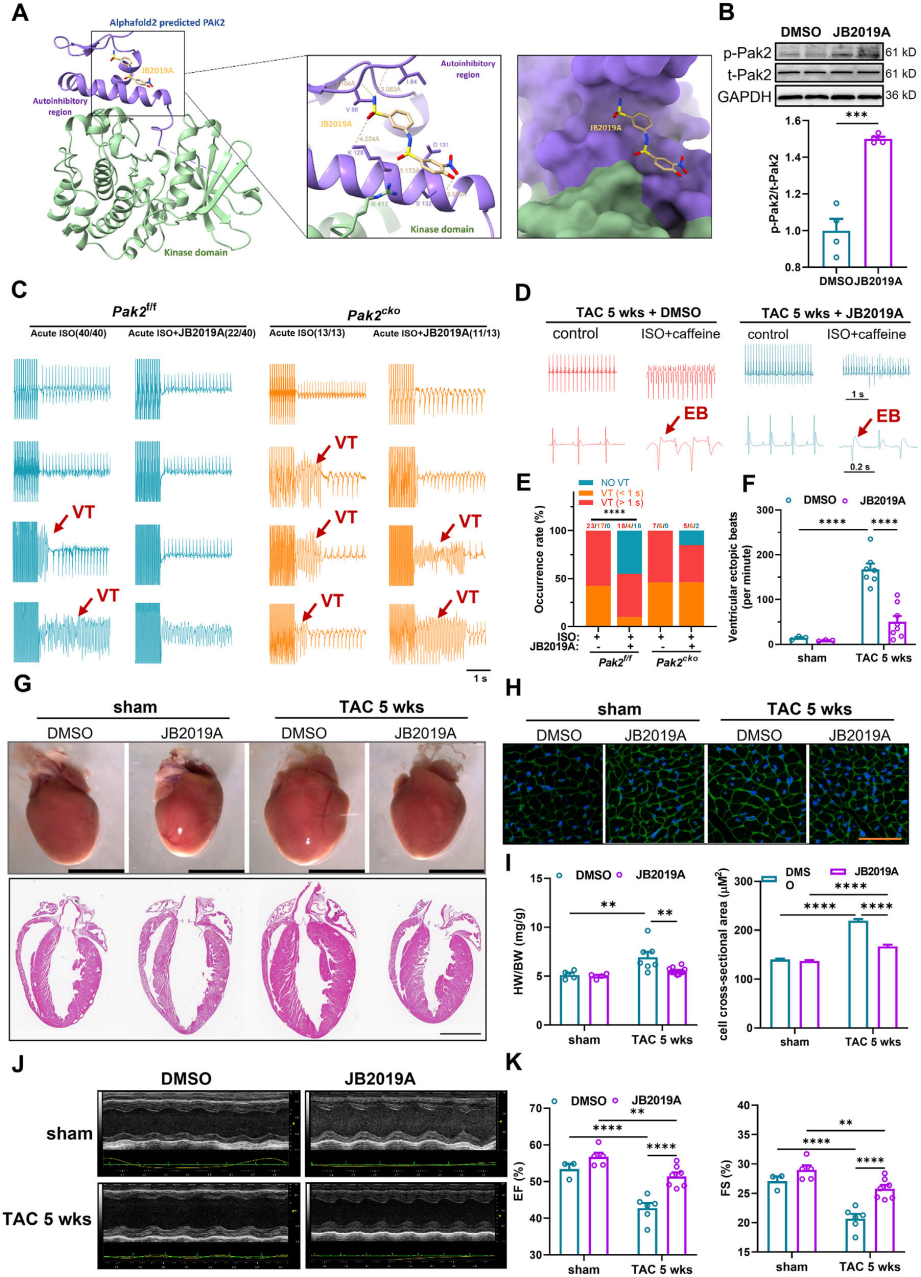
Pak2 activator JB2019A attenuates TAC‐induced cardiac hypertrophy and susceptibility to ventricular arrhythmia. WT mice were subjected to sham or TAC surgery for 5 weeks. DMSO or JB2019A (80 mg kg^−1^/d, i.p.) was injected for 4 weeks from 1 weeks after sham or TAC surgery. A) Chemical structure of Pak2 activator JB2019A. Docking model of Alphafold2 predicted Pak2 structure and its activator JB2019A. Key residues involved in JB2019A binding are labeled. Hydrogen bonds and distances are highlighted in yellow; B) Representative immunoblotting images showing p‐Pak2 protein level from WT mice with DMSO or JB2019A treatment (n = 4 mice for each group). C) Representative in vivo cardiac electrophysiological studies of Pak2^f/f^ and Pak2^cko^ mice with different frequencies of burst stimulation. D) Representative in vivo electrophysiological recordings from WT mice at 5 weeks after sham or TAC surgery. Cardiac arrhythmia was induced with isoproterenol (2 mg kg^−1^) and caffeine (160 mg kg^−1^). Arrows indicate the ventricular ectopic beats (EB). Scale bar: 1 s or 0.2 s. E) statistical graph showing the VT occurrence of C. F) Statistical graph of ventricular ectopic beats in the four groups of mice injected with isoproterenol and caffeine. G) Representative anatomic images and cardiac longitudinal morphology of WT heart at 5 weeks after sham or TAC surgery with DMSO or JB2019A treatment. Scale bar, 5 mm (Upper panel). Scale bar, 2.5 mm (Lower panel). H) WGA staining of heart tissue from indicated groups. Scale bar, 50 µm. I) HW/BW (mg/g) ratio and cell cross‐sectional area of WT heart at 5 weeks after sham or TAC surgery with DMSO or JB2019A treatment (n = 4‐7 mice). J) Echocardiographic analysis of WT heart at 5 weeks after sham or TAC surgery with DMSO or JB2019A treatment. K) Quantification of echocardiography parameters in J (n = 3‐7 mice). *p < 0.05, **p < 0.01, ***p < 0.001, ****p < 0.0001.

**Figure 9:**
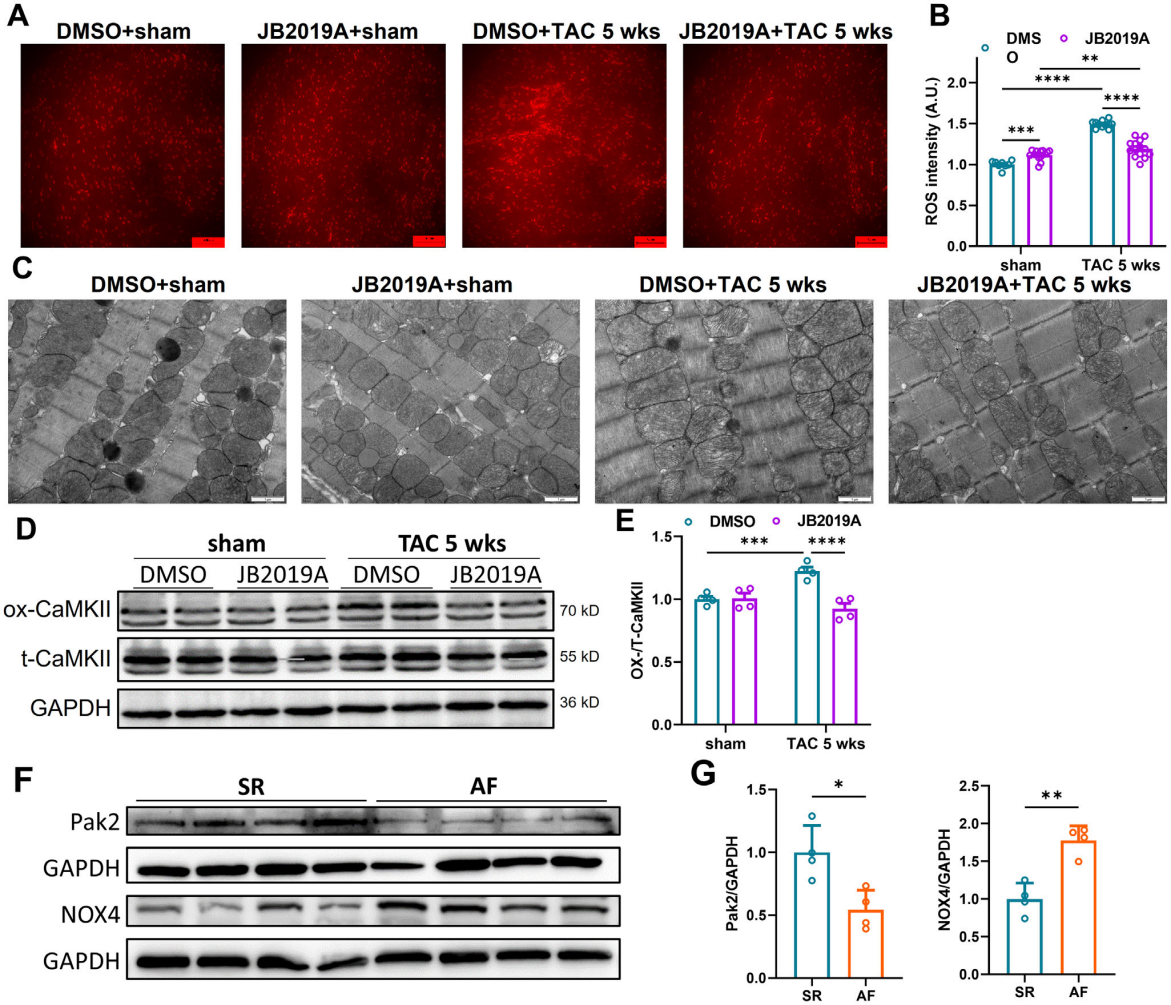
Pak2 activator JB2019A attenuates TAC‐induced cardiac oxidative stress and abnormalities in mitochondrial structure. A) Representative fluorescence images shows ROS levels of WT mice 5 weeks after sham or TAC surgery with DMSO or JB2019A treatments. Scale bar 100 µm. B) Quantification of A. C) Representative transmission electron microscopy images of WT mice at 5 weeks sham or TAC surgery with DMSO or JB2019A treatments. Scale bar 1 µm. D,E) Representative immunoblotting images and statistical graph showing ox‐CaMKII and t‐CaMKII protein expressed 5 weeks after sham or TAC surgery with DMSO or JB2019A treatments (n = 4 mice for each group). F) Representative immunoblotting images showing Pak2 and NOX4 protein levels in the myocardium of control patients and patients with atrial fibrillation. G) Quantification of F. *p < 0.05, **p < 0.01, ***p < 0.001, ****p < 0.0001.

To investigate the therapeutic viability of Pak2 as a novel therapeutic target for cardiac hypertrophy and arrhythmias, we developed the small molecule Pak2 activator JB2019A. This demonstrated a dose‐dependent Pak2 activation (**Figure** **8**A). To better understand the binding pose of JB2019A toward Pak2, we applied Autodock Vina to dock JB2019A into the predicted Pak2 structure generated by Alphafold2.^[^ ^19^ ^]^ This suggested that JB2019A binds to a site within the auto‐inhibitory domain (AID) of Pak2 (Figure 8A). This suggests that JB2019A induces an allosteric conformational change in Pak2, stabilizing its active form. Western blotting further confirmed increased phosphorylated Pak2 expression upon treatment with JB2019A, indicating higher Pak2 activation (*P* < 0.001, Figure 8B). The increased activation of Pak2 by JB2019A reduced arrhythmia and cardiac remodeling in both acute adrenergic and chronic TAC stress conditions (Figures 8 and **9**). With acute isoproterenol challenge, JB2019A decreased VT occurrences (55%, 22/40, *P* < 0.001) in *Pak2* ^f/f^, while having a minimal effects (to 84.62%, n = 11/13, *P* > 0.05) in *Pak2* ^cko^ mice (Figure 8C–F).

In WT hearts, TAC challenge increased incidences of ventricular ectopic beats from 14.33 ± 2.33 to 167.29 ± 13.33 (*P* < 0.001), but JB2019A then alleviated this effect with a decreased 50.38 ± 12.89 (*P* < 0.001) incidence. JB2019A also ameliorated TAC‐induced cardiac hypertrophy (Figure 8G–I): TAC for 5 weeks increased cardiomyocyte cross‐sectional areas and HW/BW ratios; JB2019A inhibited this effect. In contrast, JB2019A alone in the absence of TAC challenge did not affect the cardiomyocyte cross sectional areas and HW/BW ratios. Echocardiographic analysis revealed that JB2019A rescued TAC‐induced cardiac dysfunction (Figure 8J,K). JB2019A treatment also reduced oxidative stress, decreasing ROS levels and mitochondrial structural changes (Figure 9A–C). It inhibited TAC induced increments in ox‐CaMKII (Figure 9D,E); such an inhibition of the CaMKII pathway might protect against oxidative stress, including mitochondrial damage, providing a potential anti‐arrhythmic target (Figure 9A–E).

Finally, we examined Pak2 and NOX4 expression in human cardiac tissue (Figure 9F,G). Pak2 was reduced and NOX4 increased in atrial appendage tissue from cardiopulmonary bypass patients with atrial fibrillation compared to patients with sinus rhythm.

## 3 Discussion

Malignant ventricular arrhythmias associated with structural and electrical myocardial remodeling in response to myocardial stress, importantly contribute to incidences of cardiovascular death.^[^ ^2^ ^]^ They constitute a significant public health challenge, yet relatively little is understood of the underlying disease pathophysiology.^[^ ^3^, ^4^ ^]^ Previous human and animal evidence had implicated an associated mitochondrial dysfunction in such arrhythmogenesis.^[^ ^5^, ^6^ ^]^ In particular, associated ATP deficiencies and increased production of reactive oxygen species (ROS) have been implicated in a pro‐arrhythmic cellular and ionic channel malfunction.^[^ ^6^, ^7^ ^]^

Of p21‐activated kinases, previous studies implicated Pak1/Akt/eNOS signaling in a protection of cardiomyocytes against ischemic cell injury.^[^ ^20^ ^]^ The less extensively studied Pak2 is mostly localized near endoplasmic reticulum (ER) and mitochondria. Recent reports had implicated cardiac‐specific *Pak2* deletion in defective ER stress responses, cardiac dysfunction, and cardiomyocyte death with tunicamycin‐induced ER stress or pressure overload. Contrastingly, Pak2 activation produced by genetic overexpression or viral gene delivery cardioprotected against such effects.^[^ ^15^, ^16^ ^]^ The present studies go on to investigate Pak2 as a similarly promising therapeutic target for ameliorating ventricular tachyarrhythmia.^[^ ^6^, ^7^ ^]^ It traces such actions to alterations in ion channel function, intracellular Ca^2+^ homeostasis at the cellular, indices of cardiac contractile function at the whole heart,^[^ ^15^ ^]^ and mitochondrial and energetic signaling at the organelle and molecular levels for the first time.

Our study provides several lines of evidence implicating Pak2 as an anti‐oxidative stress kinase acting on ventricular electrophysiology and Ca^2+^ handling. Cardiac Pak2 deficiency in mice increased ventricular arrhythmic susceptibility and disrupted cellular Ca^2+^ handling under acute isoproterenol induced adrenergic stress or TAC‐induced chronic pressure overload. *Pak2* ^cko^ and *Pak2* ^f/f^ mice exhibited similar baseline conduction of cardiac electrical activity. However, both the acute and chronically stressed *Pak2* ^cko^ mice showed increased incidences of ventricular tachyarrhythmias (Figures 1 and 2; Figure S2 and Table S3, Supporting Information). The latter was prevented by both Pak2 overexpression and Pak2 activation by the small molecule Pak2 activator JB2019A (Figures 3 and 8). These findings implicate Pak2 as a potential therapeutic target for preventing ventricular arrhythmia.

Possible causal mechanisms underlying the increased arrhythmic susceptibility in Pak2 deficiency were further studied by high‐resolution optical mapping of Ca^2+^ transients (CaT) and membrane potentials (*V_m_*) in intact perfused hearts, and confocal microscopy line scan optical imaging and electrophysiological studies in isolated cardiomyocytes. Studies using both acutely and chronically challenged *Pak2* ^cko^ hearts demonstrated (Figure 2; Figure S2, Supporting Information) disturbed Ca^2+^ handling, including increased CaT alternans and systolic Ca^2+^ leaks, and abnormal AP waveforms. These disturbances likely contribute to the formation of re‐entry and onset of arrhythmias. Thus, Pak2 could play a vital role in maintaining the integrity of the cardiac electrical conduction system and Ca^2+^ homeostasis under both acute and chronic hypertrophic stress conditions.

Consistent with its homologies with Pak1, Pak2 also appeared to reduce oxidative stress. Previous reports had described Pak1‐mediated attenuation of NCX‐dependent Ca^2+^ overload that prevented triggered arrhythmic activity by suppressing NOX2‐dependent ROS production.^[^ ^21^ ^]^ Our investigations here implicated Pak2 in maintaining mitochondrial function. Both isoproterenol and TAC challenged *Pak2* ^cko^ hearts showed increased ROS production. In parallel with this, hypertrophic *Pak2* ^cko^/TAC hearts exhibited a more pronounced downregulation of mitochondrial respiratory chain complex I‐V proteins and their phosphorylation levels compared to *Pak2* ^f/f^/TAC hearts. These changes were accompanied by mitochondrial structural damage and reduced ATP production (Figures 5 and 6). In contrast, both Pak2 overexpression and JB2019A administration ameliorated the TAC‐induced mitochondrial structural changes and inhibited the rise in ROS levels following both acute isoproterenol and chronic TAC challenge (Figure 7, 8, 9). These findings implicate Pak2 kinase activation as a potential novel therapeutic target for cardiac hypertrophy and its arrhythmias.

Proteomics and phosphoproteomic studies showed that Pak2 deficiency associated *Pak2* ^cko^/TAC hearts with increases, particularly in mitochondrial related, proteins. KEGG and GO enrichment analysis implicated oxidative phosphorylation pathways: *Pak2* ^cko^/TAC hearts particularly showed decreased expression of ETC complexes, increased ROS release, inhibited complex I and ATP production, and abnormal mitochondrial structure.

In parallel with the increased ROS levels*, Pak2* ^cko^/TAC hearts showed increased NOX4 but not NOX2 levels, compatible with their increased ROS production. Similarly, cardiac tissue from AF patients (Figure 9), showed a Pak2 downregulation and NOX4 upregulation implicating these changes in human atrial fibrillation. Similarity, both isoproterenol and TAC challenged *Pak2* ^cko^ hearts showed increased oxidative and phosphorylated CaMKII levels reflecting CaMKII activation. These changes were alleviated in *Pak2* ^ctg^ mediated Pak2 overexpression or with Pak2 activation with the agonist JB2019A in WT.

Such findings suggest that Pak2 could act on targets involving established effects of increased ROS whether direct, or indirectly through oxidizing or directly enhancing CaMKII signaling. These may phosphorylate and activate Ca^2+^‐homeostatic proteins such as cardiac ryanodine receptors or cardiac SERCA,^[^ ^22^ ^]^ or be involved in ion channel remodeling. The resulting dysregulation of calcium handling and ion channel function could ultimately lead to development of cardiac arrhythmias.^[^ ^22^, ^23^ ^]^ Here, Pak2 activation would appears to counteract the detrimental effects of oxidative stress on CaMKII signaling, restoring normal calcium homeostasis and ion channel function. In recent modernized classifications of antiarrhythmic drugs such a potential novel therapeutic target would then add to available upstream Class VII modulators of longer‐term myocardial remodeling, complementing its classes 0‐VI of short acting antiarrhythmic drugs directed at specific ion channels.^[^ ^24^ ^]^

## 4 Conclusion

In summary, our findings identify Pak2 as a novel regulator of ROS‐induced CaMKII activation and abnormal Ca^2^⁺ dynamics in stressed hearts. CaMKII activation is likely driven by NOX4‐mediated mitochondrial oxidative stress, particularly excessive ROS production within the mitochondria. Thus, Pak2's regulation of ROS production and Ca^2^⁺ homeostasis may represent a therapeutic target for alleviating cardiac ventricular arrhythmias associated with adrenergic stress and pathological hypertrophy.

## 5 Experimental Section

All animal experiments were performed on mice in accordance with the NIH Guide for the Care and Use of Laboratory Animals and approved by the Animal Care and Use Committee of the Southwest Medical University, Sichuan (China) (No: 20160930) conforming with national guidelines under which the institution operates.

### Animal Breeding

All mice including mutant and wild‐type (WT) littermates used in this study were maintained in a specific pathogen‐free (SPF) animal center at constant temperature (25–27 °C) and humidity (45%–50%), on a 12 h/ 12 h light/dark cycle at the Southwest Medical University, Peoples Republic of China. Mice had free access to sterilized drinking water and food pellets.

### Generation of Pak2^f/f^ and Pak2^cko^ Mouse Models

The *Pak2* ^f/f^ mice were homozygous *Pak2* flox mice with two *LoxP* sites flanking exon 2 of *Pak2* constructed using the CRISPR/Cas9 technique by the Shanghai Biomodel Organism Science & Technology Development Co., Ltd. (Shanghai, China) (See the Data supplement and Figure S1, Supporting Information for details). Cardiomyocyte‐specific *Pak2* knockout mice (*Pak2* ^cko^) were generated from the *Pak2* ^f/f^ mice mated with mice expressing cyclization recombination enzyme (Cre) under the α‐myosin heavy chain (α‐MHC) promoter. The genotypes for the *Pak2* ^f/f^ and *Pak2* ^cko^ mice were verified using PCR (Figure S1, Supporting Information). The primers are shown in Table S1 (Supporting Information). The efficiency of the Pak2 knockouts was confirmed by Western blotting (Figure S1, Supporting Information).

### Generation of Pak2^ctg^ Mouse Models

The Rosa26^CAG‐LSL‐Pak2^ mice were heterozygous conditional *Pak2* overexpression mice in which CAG‐LSL‐Pak2‐WPRE‐polyA was inserted at the site of Gt (ROSA) 26S or (ENSMUSG00000086429) (Rosa 26) using the CRISPR/Cas9 technique by the Shanghai Biomodel Organism Science & Technology Development Co., Ltd. (Shanghai, China) (See Data supplement and Figure S1, Supporting Information for details). Cardiomyocyte‐specific *Pak2* over‐expression mice (*Pak2* ^ctg^) were generated from the Rosa26^CAG‐LSL‐Pak2^ mice mated with mice expressing cyclization recombination enzyme coupled with ERT (Cre‐ERT) under a α‐myosin heavy chain (Myh6) promoter. The Rosa26^CAG‐LSL‐Pak2^ mice were used as *Pak2* ^WT^ controls. Genotypes for the *Pak2^c^* ^tg^ mice were verified using PCR (Figure S1, Supporting Information). The primers were shown in Table S1 (Supporting Information). The efficiency of Pak2 overexpression was also confirmed using Western blotting (Figure S1, Supporting Information).

### Experimental Groups

Studies involving acute isoproterenol induced adrenergic stress compared results from test and control mice both not subject to any surgical procedures. Those involving chronic TAC‐induced hypertrophic (for 5 weeks or 7 weeks in studies of *Pak2* ^cko^ and *Pak2* ^ctg^ respectively) stress utilized mice subject to respective TAC and sham procedures. Of experimental groups, the experiments compared *Pak2* ^cko^ with *Pak2* ^f/f^ genotypes, apart from the comparisons of *P*ak2^ctg^ with *Pak2* ^WT^ (Rosa26^CAG‐LSL‐Pak2^) hearts. Studies testing the effects of JB2019A, used *Pak2* ^cko^ and *Pak2* ^f/f^ mice both not subject to any surgical procedures in testing the effects of acute isoproterenol induced adrenergic stress. WT mice were used to test the effect of JB2019A on chronic TAC induced hypertrophic stress.

### Surface ECG Recording in Intact Animals

Experiments on intact mice were performed under 1%–2% isoflurane anesthesia using a gas anesthesia machine (RWD Life Science, Shenzhen City, Guangdong Province, China). In vivo surface ECG monitoring to assess for ventricular tachycardia with regular waveforms (VT), or arrhythmia with irregular fibrillating waveforms (VF) used multichannel recording (MP150, BIOPAC Systems Inc, USA). Cardiac stimulation used electrodes directly placed on the cardiac surface through a thoracotomy connected to a stimulator (SEN‐7203, Nihon Kohden, Japan). A burst pacing protocol applied series of 50 (for *Pak2* ^cko^ mice) or 100 (for *Pak2* ^ctg^ mice) stimuli with successive cycle lengths (CLs) of 90, 70, 50, and 30 ms, before and following an intraperitoneal (i.p.)_isoproterenol (10 mg kg^−1^) injection to increase arrhythmic susceptibility. Additionally, animals in the TAC group were exposed to isoproterenol (2 mg kg^−1^, i.p.) and caffeine (160 mg kg^−1^, i.p.) to test for induced ventricular arrhythmia in an absence of applied stimulation as previously described.^[^ ^25^ ^]^

### Optical Mapping in Intact Hearts

Electrophysiological function in intact isolated hearts was assessed using the optical mapping system equipped with an EMCCD camera as previously described.^[^ ^26^ ^]^ Briefly, each mouse was anesthetized with 2%–5% isoflurane using the gas anesthesia machine. 10 min following an intraperitoneal heparin (3.5 U g^−1^) injection, the heart from the adult mouse was quickly removed. The isolated, spontaneously beating, heart was placed in physiological Tyrode solution (in mM) (128 NaCl, 20 NaHCO_3_, 1.18 NaH_2_PO_4_, 1.05 MgCl_2_, 4.7 KCl, 11 glucose, 1.35 CaCl_2_, pH adjusted to 7.4 with NaOH) equilibrated with 95% O_2_ and 5% CO_2_. The aorta was cannulated and Langendorff‐perfused at a constant 68–74 mmHg pressure at a 1–2 ml min^−1^ flow rate at 37 °C. The heart was subsequently slowly perfused over 10 min with Tyrode solution containing 0.4 µM RH237 (Santa Cruz Biotechnology, USA). The Ca^2+^ dye Rhod‐2 AM (Thermo Fisher Scientific, UK) was administered as a 50 µl bolus (stock solution: 1 mg ml^−1^ in DMSO) over a 5 min period and recirculated for 45 min in the presence of 0.5 mM probenecid. After dye loading, the spontaneously beating hearts were moved to a special chamber for optical mapping under an upright microscope equipped with a high‐speed EMCCD camera (Evolve 512, Photometrics, Tucson, AZ, United States). The excitation light was provided by a four light emitting diode MacroLED lamps with 525 nm (Cairn Research, UK) for excitation of RH237 and 530 nm LEDs for excitation of Ca^2+^‐sensitive dye Rhod‐2. Data was acquired at 1000 frames s^−1^. Recorded image files were uploaded into ElectroMap optical mapping analysis open source software.^[^ ^26^, ^27^ ^]^ To explore for ventricular arrhythmic tendency the burst pacing procedure was applied at the apexes of the Langendroff‐perfused hearts using pulse cycle lengths (PCL) from 100 to 20 ms.

### Echocardiographic Analysis in Intact Hearts

Mice were terminally anesthetized with 2%–5% isoflurane using the gas anesthesia machine. Transthoracic M‐mode echocardiographic recordings used the Vevo3100 micro‐ultrasound imaging system (Fujifilm VisualSonics Inc., Canada) following manufacturer's instructions. Three measurements taken at end‐systole (s) and end‐diastole (d) were averaged to calculate corresponding values of intraventricular septal thickness. Ejection fraction (EF) and fractional shortening (FS) were also obtained from the recorded measurements.

### Hematoxylin and Eosin (H & E) Staining

Hearts removed from mice terminally anesthetized with 2%–5% isoflurane using the gas anesthesia machine were transferred to 4% polyformaldehyde for fixation. Cardiac slice and haematoxylin and eosin (H & E) staining were performed following our previous protocol.^[^ ^28^ ^]^ Images were acquired using a BX63 automated microscope (Olympus, Japan) to scan the entire film under the ×20 objective lens automatically. Mean cross‐sectional areas were calculated through ≈200 randomly selected cardiomyocytes measured using Image J software.

### Transmission Electron Microscopy

Transmission electron microscopy (TEM) was performed to determine the subcellular structure of heart tissue following the different challenges (TAC or isoproterenol) used in this study as described previously.^[^ ^28^ ^]^ Briefly, cardiac tissue from the different experimental groups were fixed with 3% glutaraldehyde in phosphate buffer, then post‐fixed with 1% osmate, and dehydrated with gradient acetone. Tissues were infiltrated by a solution of epoxy resin and acetone embedded in epoxy resin. Ultra‐thin sections (50 nm) were cut and mounted on copper grids, stained with uranyl acetate and lead citrate in the dark at room temperature. Ultrastructural images were obtained under transmission electron microscopy (JEM‐1400PLUS, Japan) at 80 kV.

### Western Blotting

20 µg total protein for each lane was separated using 5% stacking gel and 10% separation gel, and transferred to a PVDF membrane (Millipore, USA). The membrane was incubated in TBST containing 5% non‐fat milk for 2 h at room temperature to block non‐specific binding and was incubated with the primary antibody (1:1, 000) overnight at 4 °C. The antibodies used in this study were listed in Table S2 (Supporting Information). The membrane was incubated with the horseradish peroxidase (HRP) conjugated goat anti‐rabbit or mouse IgG (BBI, China) secondary antibody (1:3000) for 1 h at room temperature. The membrane was incubated in chemiluminescent HRP Substrate (Millipore, USA) at room temperature for 30 s, then imaged with the Universal Hood II System (Bio‐Rad, USA).

### Isolation of Adult Cardiomyocytes and Line Scanning Laser Confocal Microscopy

Adult ventricular cardiomyocytes were isolated from a Langendorff perfused preparation. 10 min following an intraperitoneal heparin (3.5 U g^−1^) injection, the heart from the adult mouse was quickly removed. Its aorta was retrograde perfused with Ca^2+^‐free Tyrode solution comprising (in mM): NaCl 135, KCl 5.4, MgCl_2_ 1, NaH_2_PO_4_ 0.33, HEPES 10 and glucose 10 (pH to 7.3‐7.4 adjusted with NaOH). The heart was then perfused with Ca^2+^‐free Tyrode solution containing 132.6 U ml^−1^ collagenase type II (Worthington Biochemical, Lakewood, NJ, USA) for ≈15 min. The ventricle was then cut into small pieces which were filtered when the heart became flaccid. The cell suspension was then pelleted and centrifuged at 500 rpm for 30 s. The cells were then resuspended in different solutions depending on their intended use. Cells used for Ca^2+^ imaging were re‐calcified with a Ca^2+^‐containing Tyrode's solution gradient, with a final Ca^2+^ concentration of 1.8 mM. The fresh isolated single cardiomyocytes were then loaded with Fluo‐4 AM (Invitrogen, Cat. No: F14217) for intracellular Ca^2+^ staining prior to line scan using confocal microscopy (Zeiss 980, Germany). Cells used for patch clamp were stored in KB solution for later use.

### Patch Clamp Recording

Freshly isolated adult ventricular cardiomyocytes from mice in the different treatment groups were used for patch clamp electrode action potential recording using a EPC10 amplifier and PatchMaster software (Heka Elektronik, Lambrecht, Germany) as in the previous study. Current clamp mode was used to record the action potential in a whole‐cell configuration. Short current pulses (800–1000 pA, 5–10 ms) with a frequency of 1 Hz was delivered to the cell to induce action potentials. The pipette solution consisted of (in mM): K aspartate 100, KCl 40, MgCl_2_ 1.0, HEPES 5.0, K_2_‐ATP 3.0, pH 7.2 with KOH. The bath solution consisted of (in mM): NaCl 135, KCl 5.4, CaCl_2_ 1.8, MgCl_2_ 1.0, NaH_2_PO_4_ 0.33, HEPES 10, glucose 10, pH 7.3–7.4 with NaOH. The AP duration (APD) at 80% repolarization (APD_80_) was determined.

### ROS Measurement

Superoxide‐sensitive dye dihydroethidium (DHE) (Cat. No: #HY‐D0079, MedChemExpress, USA) was used for ROS measurement. Mouse cardiac tissues were embedded in Tissue‐Tek OCT (Thermo Fisher, USA). Cross‐sections (10 µm) of cardiac tissues were incubated with DHE (10 µM in 0.01% DMSO) at 37 °C for 30 min in a humidified dark chamber. Red DHE fluorescence was detected with an Olympus IX83 microscope (Olympus, Japan) at room temperature.

### ATP Level and Electron Transport Chain (ETC) Complex I Activity Measurement

The cardiac tissue was extracted and cleaned with PBS: 10 times the volume of PBS was added, the tissue was then broken up by homogenization 20 times and ultrasonication for 30 s, then centrifuged at 5000 g at 4 °C for 3 min. The supernatant was taken for measurement of mitochondrial electron transport chain (ETC) complex I activity using the ETC transport chain Complex I assay kit (#A089‐1‐1, Nanjing Jiancheng Bioengineering Institute (Nanjing, China) following the manufacturer's protocol. ATP levels were measured by the ATP Determination kit (Invitrogen, #2262562) following the manufacturer's protocol.

### Proteomics and Phosphoproteomics Array

Cardiac samples in each group were acquired and rapidly treated with liquid nitrogen and then kept at −80 °C. Each group included four individual cardiac samples. About 100 mg of cardiac tissue for each sample was extracted for protein preparation for proteomics and phosphoproteomics array constructed in Novogene Co. Ltd. (Beijing, China). TMT labelling of peptides, separation of fractions, peptide/protein identification and quantification, transition library construction, and the functional analysis of protein and differentially expressed proteins (DEP) for proteomics and phosphoproteomics were conducted according to the standard experimental and analysis protocol.

### Preparation for Ligand and Binding Modeling

Open Babel^[^ ^29^ ^]^ was used for the ligand preparation of JB2019A to create a PDBQT file that could be recognized by the docking software. The Pak2 protein structure was downloaded from the Alphafold2^[^ ^19^ ^]^ protein structure database (A0A851KSL5 (A0A851KSL5_VIDCH)). Flexible loops with a very low per‐residue confidence score were removed using UCSF ChimeraX^[^ ^30^ ^]^ to avoid the block of active sites by these loops in the docking process. The putative binding of JB2019A to Pak2 was examined using Autodock Vina^[^ ^31^ ^]^ which is an open‐source program for molecular docking. Docking models were visualized and generated using UCSF ChimeraX.^[^ ^30^ ^]^

### Clinical Samples

The human atrial appendage tissues were provided by the Affiliated Hospital of Southwest Medical University, China after approval by the local ethics committee of the Affiliated Hospital of Southwest Medical University, China (No: KY2023363). The study conformed to the principles of the Declaration of Helsinki. Human atrial appendage tissue samples were acquired from patients either in sinus rhythm or in atrial fibrillation undergoing cardiopulmonary bypass. The total protein of atrial appendage tissues was extracted and used to investigate the expression of Pak2 and other protein molecules.

### O_2_ Consumption Rates of Mitochondria from Cardiac Tissue

Mitochondria from cardiac tissue were isolated according to kit instructions (Beyotime C3606). The O_2_ consumption rates (OCR) of isolated intact mitochondria were measured with a Seahorse XFe24 analyzer (Agilent Technologies, Santa Clara, CA). Freshly isolated cardiac mitochondria were transferred to a XFe24 microplate, and the microplate filled with Mitochondrial Assay Solution (MAS, in mM): 220 mannitol, 70 sucrose, 10 MgCl_2_, 2 HEPES, 1 EGTA, 10 KH_2_PO_4_ and BSA 0.02% (w/v) at pH 7.2 to a final volume of 500 µl containing succinate (10 µM; Sigma). The OCR was measured in response to sequential injection of ADP (4 mM; Sigma), oligomycin (OA, 2.5 µg ml^−1^; MCE), trifluorocarbonyl cyanide phenylhydrazone (FCCP, 4 µM; MCE), and antimycin A (4 µM; Sigma), at 37 °C. The respiratory rates are reported as oxygen flux per mass, and all readings were normalized to µg of mitochondrial protein (pmol O_2_/min/µg protein).

### Statistical Analysis

Continuous data were presented as means ± SEM and analyzed by two‐way analysis of variance (ANOVA), assessing for independent and interacting effects of the multiple factors on each measured variate. The least significant difference (LSD) test was then used to evaluate significant differences for further multiple group comparisons. In comparisons of two groups, differences were evaluated using unpaired Student's *t*‐test. The chi‐squared (χ^2^) test was applied to analyze incidences of arrhythmias. Statistical analysis was carried out using Graphpad Prism 9.0 (Dotmatics) software. A *P‐*value of < 0.05 was considered statistically significant.

## Conflict of Interest

JB2019A was developed in ML’s group at the University of Oxford and is covered in a pending United Kingdom Patent Application No. 2412195.6 owned by University of Oxford.

## Author Contributions

Tao Li, Ting Liu, Y.W. and Y.P.L. contributed equally to this work. X.Q.T., M.L. and C.X.Z. designed the study. Tao Li1, Ting Liu, Y.X., L.Y.L., Z.L., X.L., T.T.C., X.H.O., J.B., H.L., F.Z. and Y.H. performed the experiments and acquired the data; X.Q.T., Tao Li, Ting Liu, Y.P.L., D.Z., H.L., J.Y.W. and Y.W. analyzed the data; X.Q.T., Tao Li and M.L. drafted the manuscript; Tao Li, T. Li, X.W., C.X.Z., C.L.‐H.H. and M.L. revised the manuscript; C.X.Z., X.Q.T., C.L.‐H.H. and M.L. performed final approval of the manuscript.

## Supporting information



Supporting Information

## Data Availability

The data that support the findings of this study are available from the corresponding author upon reasonable request.
