# Supplementary material for: P21‐Activated Kinase 2 as a Novel Target for Ventricular Tachyarrhythmias Associated with Cardiac Adrenergic Stress and Hypertrophy
Source: Adv Sci (Weinh). 2025 Mar 11;12(17):2411987. doi: 10.1002/advs.202411987 (PMC12061314; doi:10.1002/advs.202411987)
Supplement: Supplementary file 1 — Supporting Information [file ADVS-12-2411987-s001.pdf]

ADVANCED SCIENCE
Open Access

Supporting Information

for *Adv. Sci.*, DOI 10.1002/advs.202411987

P21-Activated Kinase 2 as a Novel Target for Ventricular Tachyarrhythmias Associated with Cardiac Adrenergic Stress and Hypertrophy

Tao Li, Ting Liu, Yan Wang, Yangpeng Li, Leiying Liu, James Bae, Yu He, Xian Luo, Zhu Liu, Tangting Chen, Xianhong Ou, Dan Zhang, Huan Lan, Juyi Wan, Yan Wei, Fang Zhao, Xin Wang, Tao Li, Christopher L.-H. Huang, Chunxiang Zhang, Ming Lei* and Xiaoqiu Tan**

Supplementary files

Supplementary Tables

Table S1. The primers for genotypes of different mouse models

Mouse model	Primer sequence (5'→3')	Size (bp)
Pak2-flox	*F1: CCCAGCACCCACAGGACAGTTTA*<br>*R1: AGAGCCTATTATCAGCATCAAGTG*	Mutant: 268 bp;<br>Wild type: 202 bp.
α-MHC-Cre	transgene identification:<br>*F1: ATGACAGACAGATCCCTCCTATCTCC*<br>*R1: CTCATCACTCGTTGCATCATCGAC*<br>internal positive control:<br>*F2: CAAATGTTGCTTGTCTGGTG*<br>*R2: GTCAGTCGAGTGCACAGTTT*	Transgene: ~300 bp;<br>Internal positive control: 200 bp
Rosa26$^{CAG\text{-}LSL\text{-}Pak2}$	*F1: TCAGATTCTTTTATAGGGGACACA*<br>*R1: TAAAGGCCACTCAATGCTCACTAA*<br>*F2: CGGTGTTTGGAAATGGATGTGGAG*<br>*R2: GGCAAGGCAGGCGGCGATGAG*	Mutant: 512 bp;<br>Wild type: 994 bp.
Myh6-Cre^{ERT}	transgene identification:<br>*F1: AGGTGGACCTGATCATGGAG*<br>*R1:ATACCGGAGATCATGCAAGC*<br>internal positive control:<br>*F2: CAAATGTTGCTTGTCTGGTG*<br>*R2: GTCAGTCGAGTGCACAGTTT*	Transgene: ~440 bp<br>Internal positive control: 200 bp

Table S2. Details of antibodies used in the present study

Protein name	Antibodies information	Company
Pak1	rabbit anti-Pak1 antibody (Cat: 2602)	Cell Signaling Technology
Pak2	rabbit anti-Pak2 antibody (Cat: 2608)	Cell Signaling Technology
Pak3	rabbit anti-Pak3 antibody (Cat: 2609)	Cell Signaling Technology
CaMKII	mouse anti-CaMKII antibody (Cat: ab22609)	Abcam
p-CaMKII	rabbit anti-CaMKII (phosphorylated) antibody (Cat: ab182647)	Abcam*
p-CaMKII	rabbit anti-CaMKII (phosphorylated) antibody (Cat: AF3493)	Affinity*
ox-CaMKII	rabbit anti-CaMKII (oxidized) antibody (Cat:	Genetex

	GTX36254)	
NOX2	rabbit anti-NOX2/gp91phox antibody (Cat: ab129068)	Abcam
NOX4	rabbit anti-NOX4 antibody (Cat: 14347-1-AP)	proteintech
NDUFB6	rabbit anti-NDUFB6 Antibody (Cat: abs132615)	Absin
NDUFA8	rabbit anti-NDUFA8 Antibody (Cat: abs134507)	Absin
NDUFS8	rabbit anti-NDUFS8 Antibody (Cat: abs139192)	Absin
GAPDH	mouse Anti-GAPDH antibody	Beyotime Biotechnology

*Use of rabbit anti-CaMKII (phosphorylated) antibody from two suppliers reflected their supplier availability in the course of the studies. Procedures using each followed the appropriate respective supplier technical specifications. Both reagent providers (Abcam and Affinity) provided reference blot bands. The reagents from Abcam (Cat: ab182647) were consistently used for the experiments on $Pak2^{cko}$ mice whose results are illustrated in Figure 6. These yielded blots with a single band as expected from the suppliers data. Reagents from Affinity (AF3493) were used in the experiments on $Pak2^{ctg}$ mice whose results are illustrated in Figure 7. Note that CaMKII (Calcium/Calmodulin-dependent Protein Kinase II) has multiple, including α, β, γ, and δ, isoforms, for which CaMKII-δ is the predominant cardiac isoform. The polyclonal Affinity antibody could then yield bands showing some non-specific signals; this is indicated in the supplier's specifications. The supplier instruction manuals provide predicted molecular weights 50, 54 and 56 kDa. Such features, shown by Figure 7D and H, have been reported on previous occasions using the same Affinity AF3493 antibody. See Supplementary file of: Jiao L, Li M, Shao Y, Zhang Y, Gong M, Yang X, Wang Y, Tan Z, Sun L, Xuan L, Yu Q, Li Y, Gao Y, Liu H, Xu H, Li X, Zhang Y, Zhang Y. lncRNA-ZFAS1 induces mitochondria-mediated apoptosis by causing cytosolic Ca^{2+} overload in myocardial infarction mice model. Cell Death Dis. 2019 Dec 9;10(12):942. doi: 10.1038/s41419-019-2136-6. PMID: 31819041; PMCID: PMC6901475.

Table S3. Statistical results of baseline parameters of *Pak2*$^{f/f}$ and *Pak2*^{cko} mice with surface ECG

groups	*HR (BPM)*	*P wave durations (ms)*	*P-R duration (ms)*	*QRS duration (ms)*	*QT duration (ms)*
Pak2$^{f/f}$ **(n=11)**	*380.17±21. 05*	*17.08±1. 51*	*47.06±3. 50*	*14.07±1. 13*	*38.65±2. 98*
Pak2^{cko} **(n=13)**	*397.62±14. 48*	*17.86±1. 89*	*44.69±3. 01*	*13.87±1. 21*	*48.24±3. 09* *

note: Unpaired Student's t-test was used. Compared with Pak2$^{f/f}$ *group,* $^{*}P < 0.05$.

Table S4. Statistical results of differential protein numbers in each comparison

Compared Samples	*Num. of Total Quant.*	*Num. of Total Sig.*	*Num. of Sig.Up*	*Num. of Sig.down*
Pak2$^{f/f}$***_TAC.vs.Pak2***$^{f/f}$***_Sham***	*4203*	*52*	*25*	*28*
Pak2^{cko}***_Sham.vs.Pak2***$^{f/f}$***_Sham***	*4203*	*149*	*82*	*67*
Pak2^{cko}***_TAC.vs.Pak2***^{cko}***_Sham***	*4203*	*806*	*388*	*418*
Pak2^{cko}***_TAC.vs.Pak2***$^{f/f}$***_TAC***	*4203*	*1029*	*598*	*431*

Note: the standard for sorting significantly differential proteins was $P \leqq 0.05$*, Fold of change (FC)* $\geqq 1.2$ *for proteomics array.*

Supplementary Figure Legends

Figure S1. Construction of cardiac-specific Pak2 knockout and overexpressing mice. ***A***, Diagram showing construction mode of cardiomyocyte-specific Pak2 knockout and over-expression mice. ***B***, Gel electrophoresis for Pak2 knockout and overexpression mice genotyping. ***C***, Representative western blotting results and quantificational statistics of Pak1-3 proteins expression from $Pak2^{f/f}$ and $Pak2^{cko}$ heart homogenates (n=4 for each group). ***D***, Representative western blotting images of Pak2 protein expression from $Pak2^{f/f}$ and $Pak2^{cko}$ in lung, brain, liver and kidney. ***E***, Representative immunoblotting images and statistical graph showing Pak2 protein in *WT* and $Pak2^{ctg}$ mice (n=4 for each group). *$P < 0.05$; **$P < 0.01$, *** $P < 0.001$. **** $P < 0.0001$.

Figure S2. Cardiac Pak2 deficiency promotes TAC-induced cardiac hypertrophy. $Pak2^{f/f}$ and $Pak2^{cko}$ mice were subjected to sham or TAC surgery for 5 weeks. ***A and B,*** Western blotting showing that Pak2 was downregulated in heart tissue from the mice with TAC surgery for 5 weeks. ***C***, Representative anatomic images, cardiac longitudinal morphology and WGA staining of heart tissues of $Pak2^{f/f}$ and $Pak2^{cko}$ heart at 5 weeks after sham or TAC surgery. Scale bar, 5 mm (*upper panel*). Scale bar, 2.5 mm (*middle panel*). Scale bar, 50 µm (*lower panel*). ***D***, Quantification of HW/BW (mg/g) and cardiomyocyte cross-sectional area of $Pak2^{f/f}$ and $Pak2^{cko}$ mice 5 weeks after sham or TAC surgery (n=8-10). ***E***, Representative immunoblotting images and quantification showing the MYH7 protein levels in $Pak2^{f/f}$ and $Pak2^{cko}$ mice 5 weeks after sham or TAC surgery (n=4). ***F***, Echocardiographic analysis of $Pak2^{f/f}$ and $Pak2^{cko}$ mice 5 weeks after sham or TAC surgery (n=9-12 for each group). *$P < 0.05$; **$P < 0.01$, *** $P < 0.001$. **** $P < 0.0001$.

Figure S3. Pak2 deficiency disrupts cardiac Ca^{2+} homeostasis. ***A***, representative APD_{80} and $CaTD_{50}$ heat maps and traces of $Pak2^{f/f}$ and $Pak2^{cko}$ heart at 5 weeks after sham or TAC surgery. ***B***, Quantification of APD_{80} and $CaTD_{50}$ from indicated groups (n=5-12). ***C***, typical AP patch clamp recordings and statistical graphs comparing APD80 in isolated single cardiomyocytes from $Pak2^{cko}$, $Pak2^{f/f}$, $Pak2^{cko}$/TAC, and $Pak2^{f/f}$/TAC hearts. ***D***, statistical graph showing Pak2

knockout increased isoproterenol-induced arrhythmias in Langendroff heart *in vitro*. ***E***, typical Ca^{2+} alternans heat maps and corresponding traces at baseline and after isoproterenol challenge in two indicated groups. *$P < 0.05$; **$P < 0.01$, *** $P < 0.001$. **** $P < 0.001$.

Figure S4. Pak2 deficiency aggravates TAC-induced abnormalities in cardiac electrophysiological activity and susceptibility to ventricular arrhythmia. ***A*,** representative AP and Ca^{2+} traces of *Pak2*$^{f/f}$ and *Pak2*^{cko} heart at 5 weeks after sham or TAC surgery. ***B*,** Statistical graphs showing the latency between V_m and Ca^{2+}. *$P < 0.05$; **$P < 0.01$, *** $P <$ 0.001.

Figure S5. Cardiac specific Pak2 overexpression attenuates TAC-induced cardiac hypertrophy and susceptibility to ventricular arrhythmia. *Pak2*^{WT} (Rosa26$^{CAG\text{-}LSL\text{-}Pak2}$*)* and Pak2 overexpression (*Pak2*^{ctg}) mice were subjected to sham or TAC operation for 7 weeks. ***A***, Representative anatomic images, cardiac longitudinal morphology and WGA staining of *Pak2*^{WT} and *Pak2*^{ctg} hearts at 7 weeks after sham or TAC surgery. Scale bar, 5 mm (*upper panel*). Scale bar, 2.5 mm (*middle panel*). Scale bar, 50 μm (*lower panel*). ***B***, Quantification of HW/BW (mg/g) and cardiomyocyte area of *WT* and *Pak2*^{ctg} mice at 7 weeks after sham or TAC surgery (n=5-10). ***C***, Echocardiographic analysis of *WT* and *Pak2*^{ctg} mice at 7 weeks after sham or TAC surgery (n=7-10). ***D***, Quantification of echocardiography parameters in C. ***E***, In vivo ECGs of *Pak2*^{WT} and *Pak2*^{ctg} with frequency-dependent pacing (PCL from 90 ms to 30 ms, 100 times for each pacing) under baseline and acute isoproterenol stress conditions. Arrows indicate the ventricular tachycardia arrhythmias (VT). Scale bar, 1 s. ***F***, Surface ECGs in vivo from a lead II in *Pak2*^{WT} and *Pak2*^{ctg} mice at 7 weeks after sham or TAC surgery injected with isoproterenol (2 mg/kg) and caffeine (160 mg/kg). Arrows indicate the ventricular ectopic beats (EB). Scale bar: 1 s or 0.2 s. ***G*** and ***H*,** Quantification of APD_{80} variance and $CaTD_{50}$ showing that Pak2 overexpression attenuates TAC-induced cardiac APD and CaTD disorder (n=4-8). *$P < 0.05$; **$P < 0.01$, *** $P < 0.001$. **** $P < 0.001$.

Figure S6. Cardiac Pak2 deficiency aggravates TAC-induced mitochondrial dysfunction

and oxidative stress. ***A***, The heat map showing the hierarchical cluster analysis of protein expression level from indicated groups. ***B***, Proteomics array from *Pak2*$^{f/f}$ and *Pak2*^{cko} mice at 5 weeks after sham or TAC surgery revealed that cardiac Pak2 knockout combined with TAC challenge induced more obvious change of protein. ***C***, Pie chart showing the subcellular location of differential protein in the comparison between the *Pak2*^{cko}/TAC and *Pak2*$^{f/f}$/TAC or *Pak2*^{cko}/sham group. ***D,*** KEGG enrichment of analysis of sequencing data between the *Pak2*^{cko}/TAC and *Pak2*^{cko}/sham group. ***E,*** KEGG enrichment of analysis of sequencing data between the *Pak2*^{cko}/TAC and *Pak2*$^{f/f}$/TAC group. ***F,*** GESA enrichment of analysis of sequencing data from indicated groups.

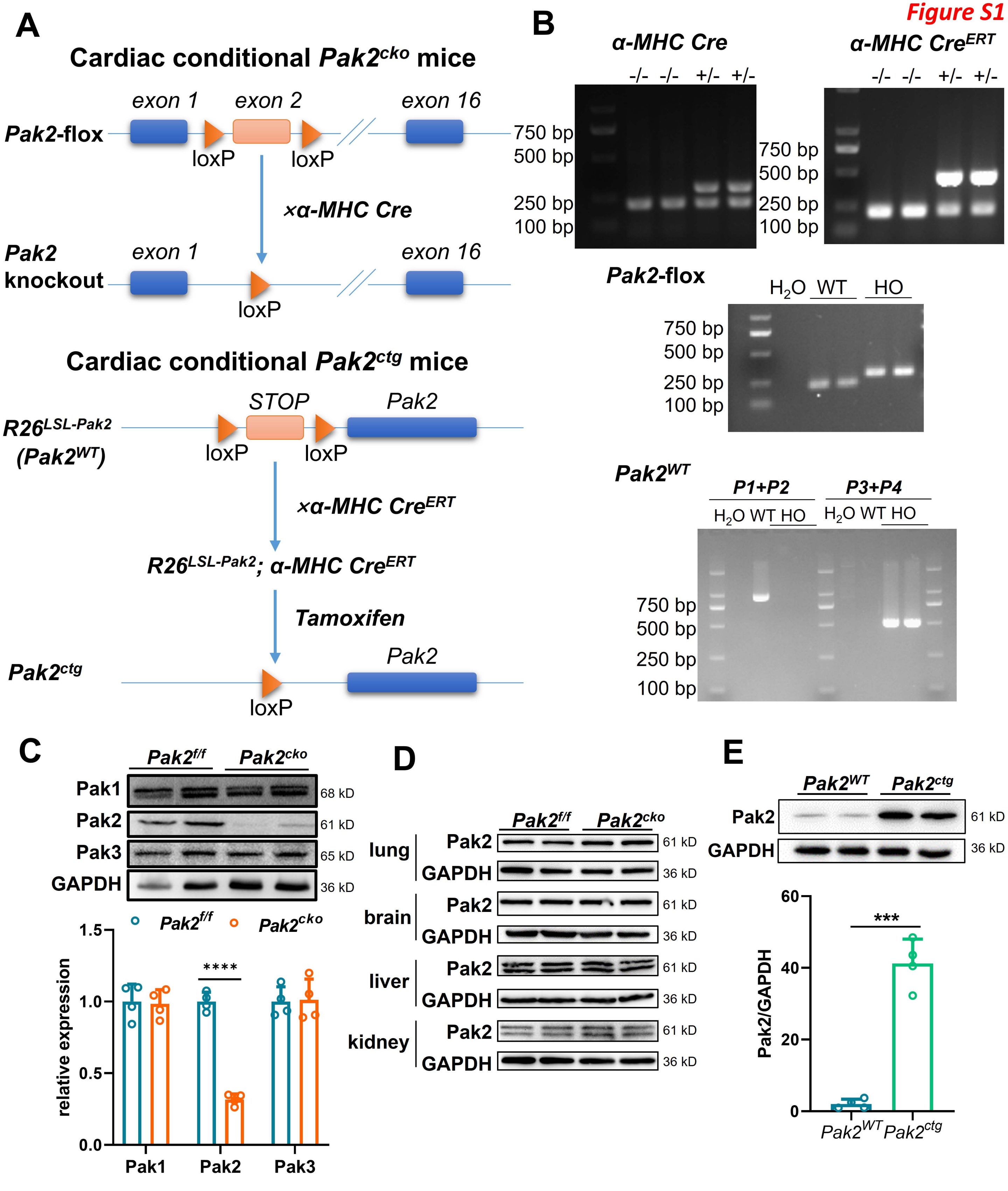

Figure S1
A
Cardiac conditional Pak2cko mice
exon 1
exon 2
exon 16
Pak2-flox
loxP
loxP
×α-MHC Cre
Pak2 knockout
exon 1
exon 16
loxP
Cardiac conditional Pak2ctg mice
STOP
Pak2
R26LSL-Pak2 (Pak2WT)
loxP
loxP
×α-MHC CreERT
R26LSL-Pak2; α-MHC CreERT
Tamoxifen
Pak2ctg
Pak2
loxP
B
α-MHC Cre
-/- -/- +/- +/-
750 bp
500 bp
250 bp
100 bp
α-MHC CreERT
-/- -/- +/- +/-
750 bp
500 bp
250 bp
100 bp
Pak2-flox
H2O WT HO
750 bp
500 bp
250 bp
100 bp
Pak2WT
P1+P2
P3+P4
H2O WT HO
H2O WT HO
750 bp
500 bp
250 bp
100 bp
C
Pak2f/f
Pak2cko
Pak1
Pak2
Pak3
GAPDH
68 kD
61 kD
65 kD
36 kD
Pak2f/f
Pak2cko
relative expression
1.5
1.0
0.5
0.0
****
Pak1
Pak2
Pak3
D
Pak2f/f
Pak2cko
lung
brain
liver
kidney
Pak2
GAPDH
61 kD
36 kD
E
Pak2WT
Pak2ctg
Pak2
GAPDH
61 kD
36 kD
Pak2/GAPDH
60
40
20
0
***
Pak2WT
Pak2ctg

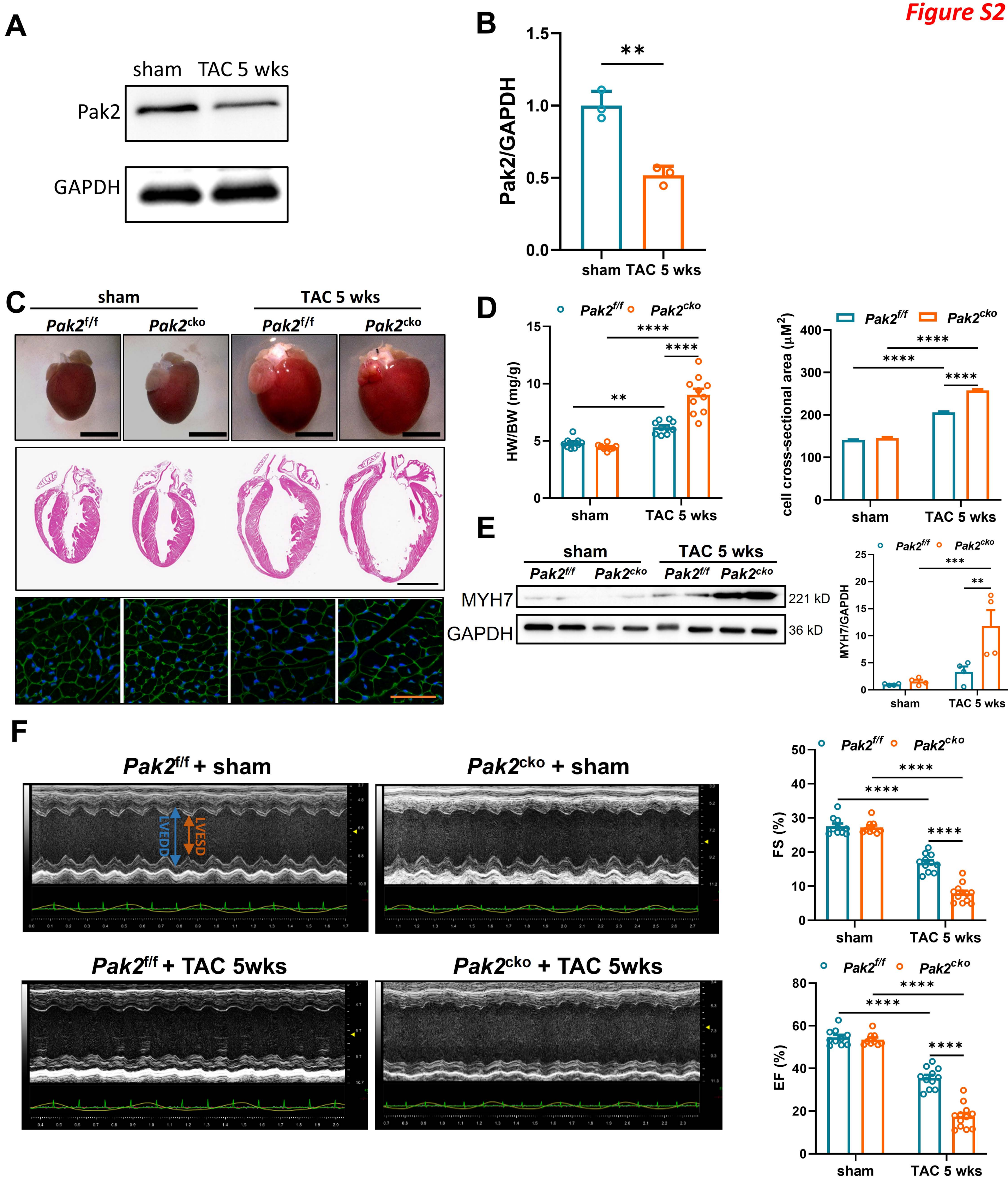
Figure S2
A
sham
TAC 5 wks
Pak2
GAPDH
B
Pak2/GAPDH
**
1.5
1.0
0.5
0.0
sham
TAC 5 wks
C
sham
TAC 5 wks
Pak2f/f
Pak2cko
D
Pak2f/f
Pak2cko
HW/BW (mg/g)
****
**
15
10
5
0
sham
TAC 5 wks
cell cross-sectional area (μM²)
400
300
200
100
0
E
MYH7
GAPDH
221 kD
36 kD
MYH7/GAPDH
***
25
20
15
10
5
0
F
Pak2f/f + sham
Pak2cko + sham
Pak2f/f + TAC 5wks
Pak2cko + TAC 5wks
LVEDD
LVESD
FS (%)
50
40
30
20
10
0
EF (%)
80
60
40
20
0

Figure S3

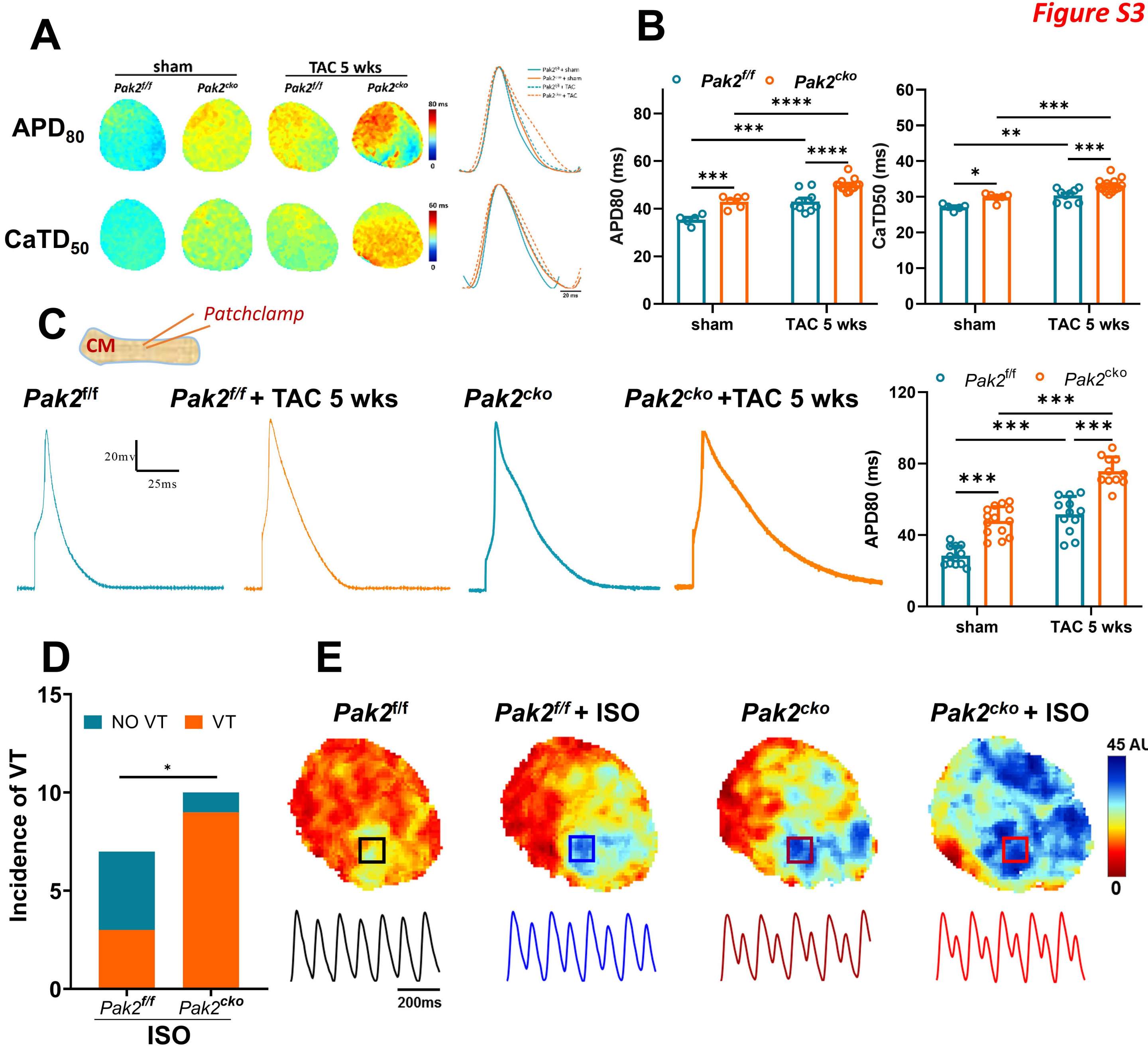
A
sham
TAC 5 wks
Pak2f/f
Pak2cko
APD80
CaTD50
80 ms
60 ms
0
20 ms
B
Pak2f/f
Pak2cko
APD80 (ms)
CaTD50 (ms)
sham
TAC 5 wks
C
Patchclamp
CM
Pak2f/f + TAC 5 wks
Pak2cko +TAC 5 wks
20mv
25ms
APD80 (ms)
D
NO VT
VT
Incidence of VT
ISO
E
Pak2f/f + ISO
Pak2cko + ISO
45 AU
200ms

Figure S4

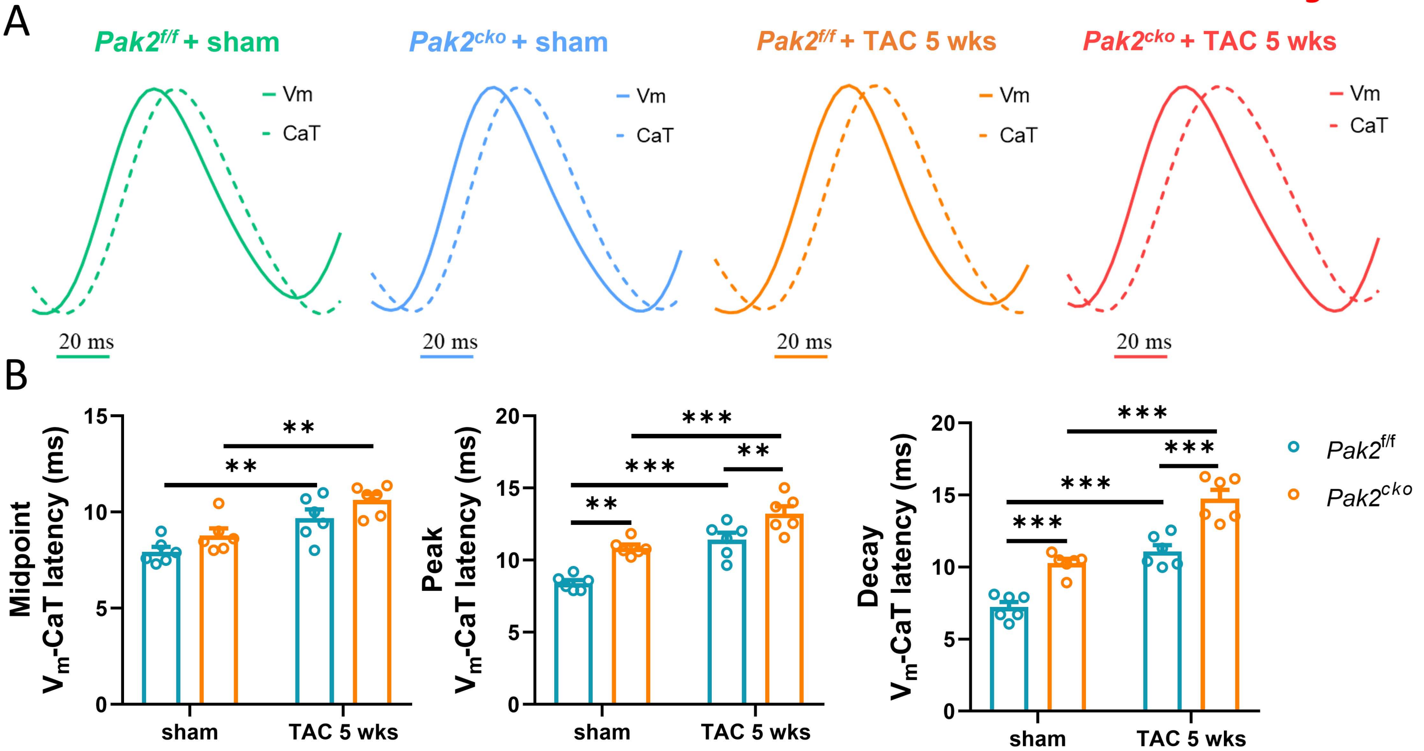
A
Pak2f/f + sham
Pak2cko + sham
Pak2f/f + TAC 5 wks
Pak2cko + TAC 5 wks
Vm
CaT
20 ms
B
Midpoint
Vm-CaT latency (ms)
Peak
Vm-CaT latency (ms)
Decay
Vm-CaT latency (ms)
sham
TAC 5 wks
**
***
Pak2f/f
Pak2cko

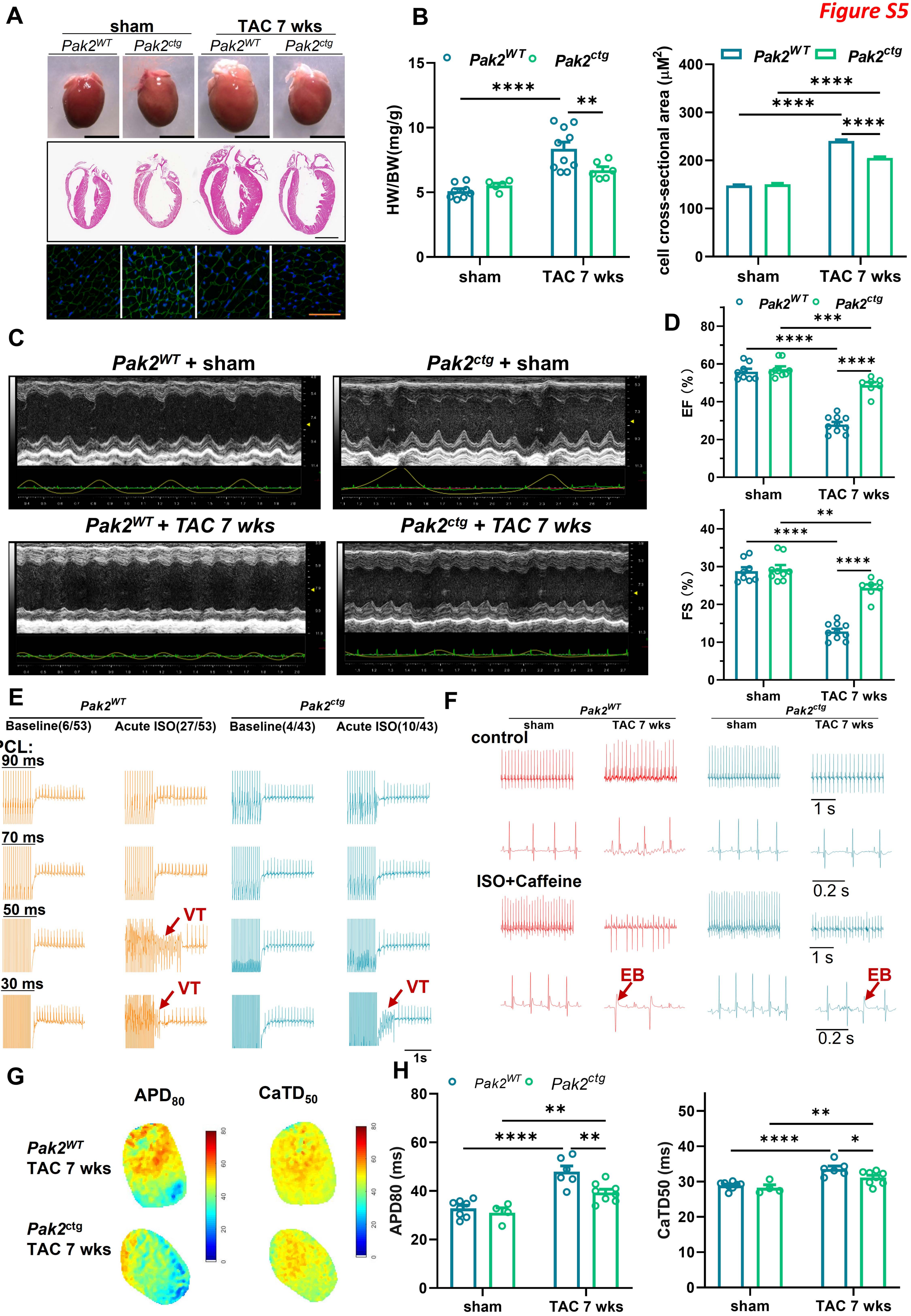

Figure S5
A
sham
TAC 7 wks
Pak2WT
Pak2ctg
B
HW/BW(mg/g)
cell cross-sectional area (μM2)
sham
TAC 7 wks
C
Pak2WT + sham
Pak2ctg + sham
Pak2WT + TAC 7 wks
Pak2ctg + TAC 7 wks
D
EF（%）
FS（%）
E
Baseline(6/53)
Acute ISO(27/53)
Baseline(4/43)
Acute ISO(10/43)
PCL:
90 ms
70 ms
50 ms
30 ms
VT
1s
F
control
ISO+Caffeine
1 s
0.2 s
EB
G
APD80
CaTD50
Pak2WT TAC 7 wks
Pak2ctg TAC 7 wks
H
APD80 (ms)
CaTD50 (ms)

Figure S6

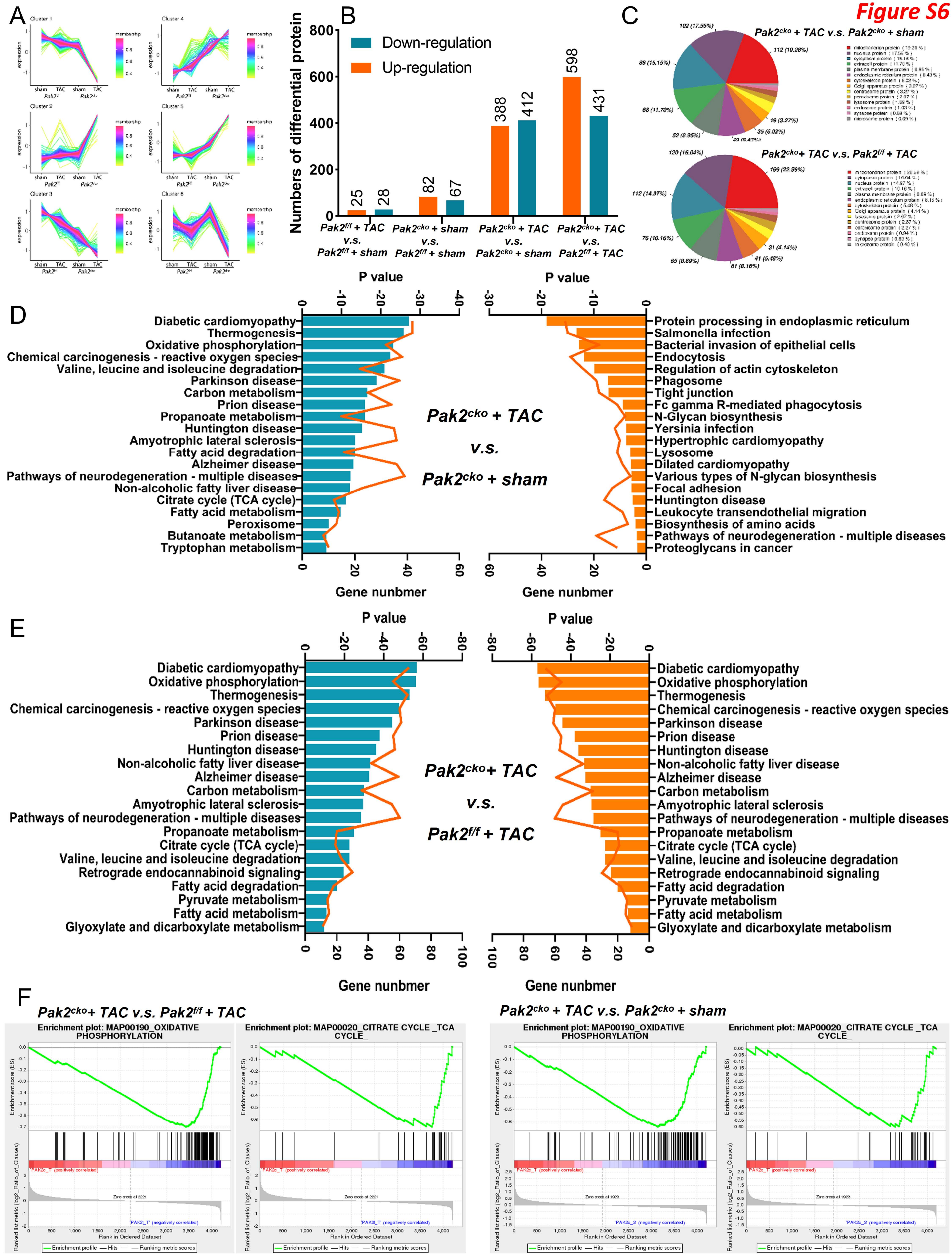
A
Cluster 1
Cluster 2
Cluster 3
Cluster 4
Cluster 5
Cluster 6
expression
membership
sham
TAC
B
Numbers of differential protein
Down-regulation
Up-regulation
25
28
82
67
388
412
598
431
Pak2f/f + TAC v.s. Pak2f/f + sham
Pak2cko + sham v.s. Pak2f/f + sham
Pak2cko + TAC v.s. Pak2cko + sham
Pak2cko+ TAC v.s. Pak2f/f + TAC
C
Pak2cko + TAC v.s. Pak2cko + sham
102 (17.56%)
112 (19.28%)
88 (15.15%)
68 (11.70%)
52 (8.95%)
49 (8.43%)
35 (6.02%)
19 (3.27%)
Pak2cko+ TAC v.s. Pak2f/f + TAC
120 (16.04%)
169 (22.59%)
112 (14.97%)
76 (10.16%)
65 (8.69%)
61 (8.16%)
41 (5.48%)
31 (4.14%)
D
P value
Gene nunbmer
Pak2cko + TAC
v.s.
Pak2cko + sham
Diabetic cardiomyopathy
Thermogenesis
Oxidative phosphorylation
Chemical carcinogenesis - reactive oxygen species
Valine, leucine and isoleucine degradation
Parkinson disease
Carbon metabolism
Prion disease
Propanoate metabolism
Huntington disease
Amyotrophic lateral sclerosis
Fatty acid degradation
Alzheimer disease
Pathways of neurodegeneration - multiple diseases
Non-alcoholic fatty liver disease
Citrate cycle (TCA cycle)
Fatty acid metabolism
Peroxisome
Butanoate metabolism
Tryptophan metabolism
Protein processing in endoplasmic reticulum
Salmonella infection
Bacterial invasion of epithelial cells
Endocytosis
Regulation of actin cytoskeleton
Phagosome
Tight junction
Fc gamma R-mediated phagocytosis
N-Glycan biosynthesis
Yersinia infection
Hypertrophic cardiomyopathy
Lysosome
Dilated cardiomyopathy
Various types of N-glycan biosynthesis
Focal adhesion
Huntington disease
Leukocyte transendothelial migration
Biosynthesis of amino acids
Pathways of neurodegeneration - multiple diseases
Proteoglycans in cancer
E
P value
Gene nunbmer
Pak2cko+ TAC
v.s.
Pak2f/f + TAC
Diabetic cardiomyopathy
Oxidative phosphorylation
Thermogenesis
Chemical carcinogenesis - reactive oxygen species
Parkinson disease
Prion disease
Huntington disease
Non-alcoholic fatty liver disease
Alzheimer disease
Carbon metabolism
Amyotrophic lateral sclerosis
Pathways of neurodegeneration - multiple diseases
Propanoate metabolism
Citrate cycle (TCA cycle)
Valine, leucine and isoleucine degradation
Retrograde endocannabinoid signaling
Fatty acid degradation
Pyruvate metabolism
Fatty acid metabolism
Glyoxylate and dicarboxylate metabolism
F
Pak2cko+ TAC v.s. Pak2f/f + TAC
Pak2cko + TAC v.s. Pak2cko + sham
Enrichment plot: MAP00190_OXIDATIVE PHOSPHORYLATION
Enrichment plot: MAP00020_CITRATE CYCLE _TCA CYCLE_
Enrichment score (ES)
Ranked list metric (log2_Ratio_of_Classes)
Rank in Ordered Dataset
Zero cross at 2221
Zero cross at 1923
Enrichment profile
Hits
Ranking metric scores
